# *Chlamydia trachomatis* Strain Types Have Diversified Regionally and Globally with Evidence for Recombination across Geographic Divides

**DOI:** 10.3389/fmicb.2017.02195

**Published:** 2017-11-13

**Authors:** Vitaly Smelov, Alison Vrbanac, Eleanne F. van Ess, Marlies P. Noz, Raymond Wan, Carina Eklund, Tyler Morgan, Lydia A. Shrier, Blake Sanders, Joakim Dillner, Henry J. C. de Vries, Servaas A. Morre, Deborah Dean

**Affiliations:** ^1^International Agency for Research on Cancer, World Health Organization, Lyon, France; ^2^Karolinska Institute, Stockholm, Sweden; ^3^North-Western State Medical University named after I.I. Mechnikov, St. Petersburg, Russia; ^4^UCSF Benioff Children’s Hospital Oakland Research Institute, Oakland, CA, United States; ^5^Laboratory of Immunogenetics, Department of Medical Microbiology and Infection Control, VU University Medical Center, Amsterdam, Netherlands; ^6^Department of Pediatrics, Boston Children’s Hospital, Boston, MA, United States; ^7^Center for Infection and Immunology Amsterdam, Academic Medical Center, Amsterdam, Netherlands; ^8^Department of Dermatology, Academic Medical Center, Amsterdam, Netherlands; ^9^STI Outpatient Clinic, Public Health Service of Amsterdam (GGD Amsterdam), Amsterdam, Netherlands; ^10^Institute of Public Health Genomics, Department of Genetics and Cell Biology, Research Institute GROW (School for Oncology and Developmental Biology), Faculty of Health, Medicine and Life Sciences, University of Maastricht, Maastricht, Netherlands; ^11^Department of Bioengineering, University of California, Berkeley, Berkeley, CA, United States; ^12^Department of Bioengineering, University of California, San Francisco, San Francisco, CA, United States; ^13^Departments of Medicine and Pediatrics, University of California, San Francisco, San Francisco, CA, United States

**Keywords:** *Chlamydia trachomatis*, MLST, recombination, global diversification, allele mixing, reassortment

## Abstract

*Chlamydia trachomatis* (*Ct*) is the leading cause of bacterial sexually transmitted diseases worldwide. The *Ct* Multi Locus Sequence Typing (MLST) scheme is effective in differentiating strain types (ST), deciphering transmission patterns and treatment failure, and identifying recombinant strains. Here, we analyzed 323 reference and clinical samples, including 58 samples from Russia, an area that has not previously been represented in *Ct* typing schemes, to expand our knowledge of the global diversification of *Ct* STs. The 323 samples resolved into 84 unique STs, a 3.23 higher typing resolution compared to the gold standard single locus *omp*A genotyping. Our MLST scheme showed a high discriminatory index, *D*, of 0.98 (95% CI 0.97–0.99) confirming the validity of this method for typing. Phylogenetic analyses revealed distinct branches for the phenotypic diseases of lymphogranuloma venereum, urethritis and cervicitis, and a sub-branch for ocular trachoma. Consistent with these findings, single nucleotide polymorphisms were identified that significantly correlated with each phenotype. While the overall number of unique STs per region was comparable across geographies, the number of STs was greater for Russia with a significantly higher ST/sample ratio of 0.45 (95% CI: 0.35–0.53) compared to Europe or the Americas (*p* < 0.009), which may reflect a higher level of sexual mixing with the introduction of STs from other regions and/or reassortment of alleles. Four STs were found to be significantly associated with a particular geographic region. ST23 [*p* = 0.032 (95% CI: 1–23)], ST34 [*p* = 0.019 (95% CI: 1.1–25)]; and ST19 [*p* = 0.001 (95% CI: 1.7–34.7)] were significantly associated with Netherlands compared to Russia or the Americas, while ST 30 [*p* = 0.031 (95% CI: 1.1–17.8)] was significantly associated with the Americas. ST19 was significantly associated with Netherlands and Russia compared with the Americans [*p* = 0.001 (95% CI: 1.7–34.7) and *p* = 0.006 (95% CI: 1.5–34.6), respectively]. Additionally, recombinant strains were ubiquitous in the data set [106 (32.8%)], although Europe had a significantly higher number than Russia or the Americas (*p* < 0.04), the majority of which were from Amsterdam [43 (87.8%) of 49)]. The higher number of recombinants in Europe indicates selective pressure and/or adaptive diversification that will require additional studies to elucidate.

## Introduction

The modern pathogenic *Chlamydiaceae* family has a rich evolutionary history, diverging from environmental *Chlamydiales* approximately seven million years ago (Horn et al., 2004). The human *Chlamydiaceae* spp. *Chlamydia trachomatis* (*Ct*) has infected human populations causing sexually transmitted diseases (STD) and the chronic ocular disease known as trachoma since the 27th century BC (Perkins and Hill, 2013). Trachoma was initially described in China and in the Eber’s Papyrus of Egypt, and subsequently spread to Europe during the Crusades (Perkins and Hill, 2013). While improvements in hygiene and sanitation have eliminated trachoma from many global populations, the disease is still endemic in many developing countries of Africa, Central and South America, the South Pacific and Asia in addition to aboriginal populations in Australia (Perkins and Hill, 2013). Presently, *Ct* is the leading cause of preventable blindness and bacterial sexually transmitted infections (STIs) worldwide (Rowley et al., 2012) with estimates of over 250 million trachoma cases and 110 million annual STI cases, according to the World Health Organization (WHO Sexually transmitted infections [STIs], 2015).

*Ct* has evolved to include 19 serological variants (serovars) based on antibody typing of the major outer membrane protein (MOMP) with over 60 *omp*A genotypes (Dean and Millman, 1997; Batteiger et al., 2014; Isaksson et al., 2016; Peuchant et al., 2016; Schillinger et al., 2016), the gold standard typing technique for all *Chlamydiaceae* spp. The serovars are designated A through K, Ba, Da, Ia, Ja, and L_1-3_, and L_2_a while the *omp*A genotypes or strains are denoted by the same or by a number or letter after the conventional serovar name (e.g., D1; Ga) for new genotypes (Batteiger et al., 2014). These strains are responsible for ocular, urogenital and rectal infections. Ocular infections include trachoma, a chronic ocular disease, and ophthalmia neonatorum (Darville, 2005), an infection acquired during passage through a *Ct* infected birth canal (WHO Sexually transmitted infections [STIs], 2015). Urogenital strains cause not only ocular infections, which usually present as unilateral conjunctivitis (Dean et al., 2008), but also can ascend from the endocervix to cause sequelae such as pelvic inflammatory disease, infertility and ectopic pregnancy (Mårdh, 2004; Blas et al., 2007; Baud et al., 2008). Rectal infections can progress to proctitis and inguinal syndrome (Sethi et al., 2009). While the later is caused primarily by the lymphogranuloma venereum strains (LGV) L_1-3_, L_2_a, L_2_b, and L_2_c, the former can be caused by most *Ct* strains, although strains B, Ba, and C are rarely detected in the urethra, endocervix or rectum (Danby et al., 2016; Labiran et al., 2016). Strain A is the only strain that is confined to the ocular mucosa (Dean, 2010).

Classification of *Ct* strains was conventionally performed by serotyping and more recently by *omp*A genotyping (Dean et al., 1992) since the organism is rarely cultured, a requirement for serotyping. Although *omp*A genotyping can be informative, the gene represents a mere 0.1% of the genome and is subject to immune selective pressure and recombination (Joseph et al., 2011, 2012). Finer, more holistic typing schemes are necessary to track recombination events, differentiate new and persistent infections (Götz et al., 2013), reinfection, transmission patterns and elucidate potential biomarkers. Three Multi Locus Sequence Typing (MLST) schemes have been developed for *Ct* (Klint et al., 2007; Pannekoek et al., 2008; Dean et al., 2009), of which only two meet the MLST criteria of using strictly housekeeping genes (Pannekoek et al., 2008; Dean et al., 2009). Our MLST scheme employs seven highly conserved housekeeping genes and successfully resolves reference and clinical *Ct* samples into LGV, trachoma, non-prevalent non-invasive urogenital, and prevalent non-invasive urogenital clonal complexes representing the respective diseases in addition to revealing evidence for recombination (Dean et al., 2009; Batteiger et al., 2014).

Partial and whole genome sequencing (WGS) have added considerably to our knowledge of the diversity of *Ct* and evidence for recombination. We initially bioinformatically identified recombination within *omp*A (Millman et al., 2001) and then among partial genome sequences of trachoma and sexually transmitted strains involving *omp*A and polymorphic membrane proteins (*pmp*) (Gomes et al., 2004, 2006, 2007). In the first publication of comparative WGS of *Ct*, we identified three major clades that were similar to the MLST disease-related clonal complexes with a subclade encompassing the trachoma strains within the non-prevalent non-invasive urogenital clade (Joseph et al., 2011). Subsequent WGS by our and other groups have substantiated these phylogenetic groupings as well as the recombinogenic nature of *Ct* (Harris et al., 2012; Joseph et al., 2012; Seth-Smith et al., 2013; Hadfield et al., 2017).

As whole genome sequencing remains cost-prohibitive for large sample sets and beyond the reach of most research laboratories, in this work, 323 *Ct* reference and clinical samples from 15 countries and 5 continents were analyzed by MLST to provide a more comprehensive analysis of the global diversification of *Ct* strain types. We included 60 new clinical samples from Amsterdam, Netherlands, eight from Boston, MA, United States, and 58 from St. Petersburg, Russia, a region that had not previously been evaluated by MLST.

## Materials and Methods

### Study Populations and Ethics

Information on populations and ethics for samples collected previously are included in the publications by Dean et al. (2009) and Batteiger et al. (2014). Russian women were enrolled in the original studies following verbal informed consent after approval by the Local Institutional Review Board at DO Ott Institute of Obstetrics and Gynecology and the Russian Academy of Medical Sciences (RAMS), St. Petersburg, Russia (Shipitsyna et al., 2007). The studies were additionally approved by the Department of Clinical Investigations and Intellectual Property, St. Petersburg Medical Academy of Postgraduate Studies (North-Western State Medical University named after I.I. Mechnikov since 2011), under the Federal Agency of Public Health and Social Development of Roszdrav, in accordance with the Declaration of Helsinki. For the 60 Dutch samples, each was obtained as previously described (Dean et al., 2009) according to the Declaration of Helsinki. The eight Boston samples were sent to Dr. Dean as de-identified samples with no trace to patient name and were not considered human subjects according to IRB at Children’s Hospital Oakland Research Institute and NIH guidelines.

For the Russian samples, endocervical swabs were obtained consecutively from January 2006 to January 2008 in two university clinics in St. Petersburg, Russia, as described elsewhere (Smelov et al., 2009). To minimize the potential for low response from the enrolled women, samples were collected without obtaining personal information. After removal of any mucopus with a cotton swab, a Dacron swab was inserted into the endocervix, rotated and placed in an empty 5 mL vials. Specimens were kept at 4°C (39°F) for up to 4 days before they were shipped to the laboratory where they were stored at 4°C (39°F). Within 1–3 days (Shipitsyna et al., 2007) all samples were tested for the presence of *Ct* by commercial NAAT assays (Shalepo et al., 2006; Smelov et al., 2009). Additionally, either a conventional PCR (Lytech, Moscow, Russia) or a real-time PCR (Central Research Institute of Epidemiology, Moscow, Russia) were used. The results were confirmed in Amsterdam, Netherlands by the commercial real-time PCR (TaqMan, Applied Biosystems, United States) (Morré et al., 1999; Smelov et al., 2009) and CE-IVD certificated Presto CT-NG Assays (Goffin Molecular Technologies, Beek, Netherlands) (de Waaij et al., 2015).

### *Ct* Reference and Clinical Samples

A total of 323 reference and clinical samples were analyzed that included 58 endocervical samples from St. Petersburg, 60 additional endocervical samples from Amsterdam and eight endocervical samples from Boston, Massachusetts; 265 were already in the MLST database^1^ including the 20 reference strains: A/SA-1, A/HAR13, B/TW5/OT, Ba/Apache2, C/TW3/OT, D/UW3/Cx, Da/TW-448, E/Bour, F/IC-Cal3, G/UW57/Cx, H/UW4/Cx, I/UW12/Ur, Ia/UW202, J/UW36/Cx, Ja/UW92, K/UW36/Cx, L_1_/440, L_2_/434, L_2a_/UW396, L_3_/404.

### *omp*A Genotyping and MLST Analysis

Genomic DNA was purified from clinical isolates and *omp*A genotyped as described previously (Dean et al., 2009; Batteiger et al., 2014). Only the samples from St. Petersburg were provided as swabs; DNA was extracted and purified for these samples using our protocol as described in Joseph et al. (2014). MLST analysis examined seven housekeeping genes: *glyA, mdhC, pdhA, yhbG, pykF, lysS*, and *leuS*, with primers as described (Dean et al., 2009) (Supplementary Table 1). All seven housekeeping genes were amplified and sequenced as described (Batteiger et al., 2014). A consensus sequence was created from the forward and reverse sequence. The genes for each of the St. Petersburg, Amsterdam and Boston samples were each concatenated and queried against the 265 samples in the MLST database in addition to including these samples in the database. Allelic numbers and STs were assigned based on this query as described previously (Batteiger et al., 2014).

### Phylogenetic Analysis and Strain Clustering

Using the concatenated sequences, dataset strain clustering and Single Nucleotide Polymorphism (SNP) analyses were performed as described (Batteiger et al., 2014). Briefly, this included visualizing clusters of related STs and non-related STs using eBURST^2^ (Feil et al., 2004). Founder STs were identified by the highest number of single locus variants (SLV) branching from that particular ST (i.e., the clonal ancestor that diversifies into other STs). Clonal complexes generated by eBURST were defined as a group of STs separated by one SLV.

Phylogenetic trees were created by Maximum likelihood using the Symmetric+GI model, which provided the best fit for the data, in the R package phangorn (Schliep, 2010) to analyze the nucleotide sequence variation between the seven MLST loci for each ST. Tree nodes were verified with 1,000 bootstrap replicates. Alternative evolutionary pathways, such as horizontal gene transfer, were analyzed with SplitsTree^3^ using the splits decomposition method as described (Dean et al., 2009; Tamura et al., 2013). In addition, the sequence for each of the seven MLST loci for a sample were compared across the dataset and to the *omp*A genotype of the same sample to determine evidence for putative recombination.

### Statistical Tests

Fisher’s exact test was performed in R^4^ to test for significant region-specific *omp*A and ST clustering; a *p*-value of <0.05 was considered significant. Confidence intervals were determined based on the method of Clopper-Pearson (Borkowf, 2006). Simpson’s Diversity Index, *D*, was calculated for the MLST data as described (Simpson, 1949; Hunter and Gaston, 1988). A *D*-value of ≥0.95 was considered ideal for molecular typing techniques (van Belkum et al., 2007). The *omp*A genotypes were excluded from the analysis. The Benjamin-Hochberg FDR method (Benjamini and Hochberg, 1995) was used to correct *p*-values for multiple comparisons.

Samples were classified as putative recombinants when the sequences of the seven gene sequences that comprise the ST or any of the seven genes were non-concordant with each other or with the *omp*A genotype of the same sample.

PROC FREQ in SAS was used to identify SNPs associated with disease phenotype and Haplotype as described previously (Dean et al., 2009). Levene’s test evaluated the variance across the dataset of the 323 samples. The Classification Index was used to determine significance of each SNP with a disease phenotype where a *p*-value of <0.05 was considered significant.

DnaSP v5.10 (Librado and Rozas, 2009) was used to calculate nucleotide (nt) and haplotype (hd) to determine the genetic diversity and differentiation for regional subgroups on the concatenated sequences of the seven MLST genes. DnaSP considers the frequency of variants (STs) present in a population and also genetic distances that separate these variants from each other. Genetic population differentiation between regional subgroups was assessed using the pairwise fixation index (Fst) in Arlequin v3.5 (Excoffier and Lischer, 2010) with significance testing by permutation.

## Results

### Characteristics and Geographic Distribution of Alleles

The characteristics of the alleles for each gene locus are shown in Supplementary Table 2. The number of alleles varied by gene locus, ranging from seven to 18, as did the number of polymorphic sites. We determined the allele frequencies by geographic region for the 78 alleles (**Table 1**). Thirty-two (41%) alleles were observed once. The highest number of unique alleles for a geographic region was in Western Europe at 16 alleles but the highest frequency was 43.3% for Russia, which was not statistically significant.

**Table 1:** Allele frequencies by geographic region by locus.

Gene locus	No. alleles	Africa *n* = 21 (%)	Western Europe *n* = 109 (%)	Russia *n* = 58 (%)	Asia *n* = 13 (%)	Americas *n* = 122 (%)	Classification index *p*-value
*glyA*	12	1 (14.29)	1 (12.84)			1 (7.37)	<0.001
			3 (50.46)	3 (62.07)	3 (30.77)	**2 (0.82)**	
		3 (80.95)	**4 (0.92)**	6 (34.48)		3 (42.62)	
		6 (4.76)	**5 (1.83)**	**10 (1.72)**	6 (7.69)		
			6 (32.11)	**11 (1.72)**	**7 (61.54)**	6 (48.36)	
			**8 (0.92)**				
			**9 (0.92)**			**12 (0.82)**	
*mdhC*	7	1 (14.29)	1 (11.93)			1 (8.19)	<0.001
		3 (76.19)	**2 (2.75)**	3 (63.79)	3 (100.00)	3 (68.85)	
		4 (4.76)	3 (70.64)	4 (34.48)			
		**5 (4.76)**	4 (12.84)	**7 (1.72)**		4 (22.95)	
			**6 (1.83)**				
*pdhA*	8	**1 (4.76)**					<0.001
		3 (95.24)	**2 (0.92)**	3 (94.83)	3 (100)	3 (97.54)	
			3 (91.74)	5 (3.45)			
			**4 (2.75)**	**8 (1.72)**		5 (1.64)	
			5 (3.67)			**6 (0.82)**	
			**7 (0.92)**				
*ybhG*	11				**1 (7.69)**		<0.001
		2 (4.76)	2 (31.19)	2 (44.83)	**4 (7.69)**	2 (43.44)	
		6 (80.96)	3 (0.92)	6 (48.28)	5 (7.69)		
		8 (14.29)	5 (1.83)	9 (5.17)	6 (76.92)	3 (0.82)	
			6 (48.62)	**11 (1.72)**		5 (9.83)	
			**7 (0.92)**			6 (37.70)	
			8 (15.60)				
			9 (0.92)			8 (7.38)	
						**10 (0.82)**	
*pykF*	10	1 (14.29)	1 (15.60)			1 (8.20)	<0.001
		3 (71.43)	6 (49.54)	6 (53.45)	3 (92.31)	**2 (2.46)**	
		6 (9.52)	7 (33.03)	7 (37.93)		**4 (0.82)**	
		7 (4.76)	**8 (0.92)**	**10 (8.62)**	7 (7.69)	**5 (0.82)**	
			**9 (0.92)**			6 (36.07)	
						7 (51.64)	
*lysS*	12		1 (6.42)	1 (8.62)		1 (7.38)	<0.001
		4 (23.81)	4 (74.31)	4 (74.14)	4 (7.69)	**2 (0.82)**	
		5 (71.43)	8 (18.35)	8 (13.79)	5 (30.77)	**3 (0.82)**	
		**7 (4.76)**	**10 (0.92)**	**11 (1.72)**		4 (73.77)	
				**12 (1.72)**	**6 (61.54)**	8 (14.75)	
						**9 (2.46)**	
*leusS*	18					**1 (2.46)**	<0.001
		2 (4.76)	3 (75.23)	3 (79.31)	2 (7.69)	3 (67.21)	
		3 (4.76)	**4 (0.92)**	6 (8.62)	7 (84.62)		
		7 (9.52)	**5 (0.92)**	8 (3.45)		6 (5.74)	
		9 (66.67)	6 (3.67)	**14 (1.72)**	**10 (7.69)**	8 (14.75)	
		11 (14.29)	8 (2.75)	**15 (1.72)**			
			11 (15.60)	**16 (1.72)**		9 (0.82)	
			**13 (0.92)**	**17 (1.72)**		**11 (7.38)**	
				**18 (1.72)**		**12 (1.64)**	
No. novel alleles per region		3 (12.5%)	16 (40%)	13 (43.3%)	5 (29.4)	13 (36.1%)	
Total no. alleles	78	24	40	30	17	36	

^∗^*Numbers are arranged vertically for each locus to represent the individual alleles (e.g., *gly*A alleles are assigned 1, 2, 3, 4, 5, 6, 7…12 because there are 12 alleles for this locus). Alleles marked in boldface are specific for a single geographic region. n, number of samples*.

### ST and *omp*A Distributions

For the 323 samples, 84 unique STs were identified (Supplementary Table 3). STs novel to the dataset were numbered consecutively in order of identification. Of those 84, 57 (67.9%) were singletons, with a relatively even distribution by geographic region (excluding Asia and Africa where the sample sizes were very small) with a higher percentage of singletons in Europe that was not significantly different (**Table 2**; *p* = 0.08). **Table 2** also shows that the percentage of unique STs per region was highly similar. However, the ST/sample ratio was significantly greater for Russian than for European and American samples (*p* < 0.009). There were also significant differences in the distribution of STs (**Table 3**). Dutch females (*n* = 79) were significantly more likely to be infected with ST23 (*p* = 0.032; 95% CI: 1–23) and ST34 (*p* = 0.02; 95% CI: 1.1–25) compared to Russian females (*n* = 58) and with ST19 (*p* = 0.001; 95% CI: 1.7–34.7) compared to American females (*n* = 108). Supplementary Table 4 shows the distribution of STs by geographic region.

**Table 2:** Strain type (ST) diversity and recombinants by geographic region.

Geographic location	Number of Samples	Number of ST’s	ST/sample ratio (95% CI)	Number ST Singletons (%)	Number Novel STs per Region (%)	Number Recombinant Samples (%)
Europe	109	28	0.26 (0.18–0.39)	17 (61)	19 (68)	49 (45)^∗∗^
Americas	122	34	0.28 (0.20–0.40)	16 (47)	24 (71)	28 (23)
Asia	13	8	0.57 (0.48–0.66)	5 (63)	8 (100)	1 (7.7)
Africa	21	7	0.35 (0.26–0.44)	5 (71)	5 (71)	8 (38.1)
Russia	58	26	0.45^∗^(0.35–0.53)	14 (54)	18 (69)	20 (34.5)
**Total**	**323**			**57**		**106 (32.8)**

^∗^*Significantly increased compared to Europe and the Americas (*p* < 0.009); Asia and Africa were excluded due to small numbers. ^∗∗^Significantly increased compared to Russia and the Americas (*p* < 0.04)*.

**Table 3:** Differences in ST distribution by geographic region.

Regions of comparison^∗^	ST	Significance
Netherlands vs. Russia	23	*p* = 0.03199 (95% CI: 1–23)
Netherlands vs. Russia	34	*p* = 0.0188 (95% CI: 1.1–25)
Netherlands vs. Americas	19	*p* = 0.000399 (95% CI: 1.7–34.7)
Americas vs. Netherlands	39	*p* = 0.03127 (95% CI: 1.1–17.8)
Russia vs. Americas	19	*p* = 0.005752 (95% CI: 1.5–34.6)

^∗^*Women were analyzed separately for the purposes of comparing similar cohorts*.

There were 26 *omp*A genotypes observed in the dataset resulting in a 3.23 lower resolution than for STs. Excluding the STDs samples from South Africa, all samples available from Asia and Africa were from trachoma patients; 1 (7.1%) of 14 was a urogenital Da *omp*A genotype (ST 37) in Asia while 1 (5.9%) of 17 was a urogenital E genotype (ST 39) in Africa.

*omp*A genotype E (*n* = 72) was the most prevalent and associated with 18 STs with an ST to sample ratio of 0.25 (Supplementary Table 3). The distribution for the remaining *omp*A genotypes in descending order was G (38; 16 STs; ratio: 0.42); D (33; 16 STs; ratio: 0.48); F (30; 5 STs; ratio: 0.17); Ia (17; 4 STs; ratio: 0.25); K (15; 7 STs; ratio: 0.47); J (13; 9 STs; ratio: 0.69); B, H and I at 12 samples each (7, 6, and 3 STs, respectively; ratios: 0.58, 0.50, and.25, respectively); C (11: 5 STs; ratio: 0.45); A (9; 4 STs; ratio: 0.44); D2 and Ja at 4 samples each (1 and 3 STs, respectively; ratios: 0.25 and 0.75, respectively); Ba, D1, E6, F4 and Ia4 at 2 samples each (1 ST for each; ratio: 0.50 for each); and Da (1; 1 ST; ratio: 0.10).

The *omp*A distribution of urogenital strains varied across Europe, the Americas, and Russia. In Europe, *omp*A genotype D was significantly more prevalent than in other geographic regions (*p* = 0.046) and more frequent than the globally prevalent genotype E. In Russia, *omp*A genotype G was significantly more prevalent than in all other regions (*p* = 0.001). In the Americas, *omp*A genotype Ia was significantly more prevalent than the other regions (*p* = 0.001). In comparing female STD cohorts, Russian women were significantly more likely to be infected with E and G (*p* = 0.001 and 0.026, respectively) while Dutch women were significantly more likely to be infected with D and I (*p* = 0.026 and 0.002, respectively).

Simpson’s Discriminatory Index was 0.98 (95% CI 0.97–0.99) for MLST and 0.70 (95% CI 0.67–0.73) for *omp*A genotyping.

Based on DnaSP, nucleotide diversity was lower in Russia (Pi = 0.00216) compared to Netherlands (Pi = 0.00321) and North America (Pi = 0.00294) (**Table 4**). However, Netherlands and North American datasets contained isolates from men and LGV samples. The phylogenetically disparate LGV samples appeared to contribute substantially to nucleotide diversity; nucleotide diversity dropped from 0.00250 to 0.00196 in Dutch women when the four LGV isolates were removed. When comparing non-LGV isolates from women in these regions, Russian women had the highest nucleotide diversity (Pi = 0.00216), followed by North American women (Pi = 0.00210), and Dutch women (Pi = 0.00196).

**Table 4:** Nucleotide and haplotype diversity.

Subgroup	*N*	Nucleotide diversity(nt)	Haplotype diversity(hd)
Russia	58	0.00216 ± 0.00007	0.917 ± 0.023
Netherlands	79	0.00321 ± 0.00030	0.879 ± 0.020
North America	116	0.00294 ± 0.00023	0.926 ± 0.012
North American Women	66	0.00210 ± 0.00009	0.925 ± 0.016
Netherlands Women	72	0.00250 ± 0.00026	0.863 ± 0.024
Netherlands Women non-LGV	68	0.00196 ± 0.00011	0.846 ± 0.026

Assessing population differentiation between regional subgroups by Fst revealed significant differences between most regional subgroup pairwise comparisons (**Table 5**). African and Asian subgroups exhibited high Fst values in all comparisons, indicating those regions were distinct from others in the dataset. This is not surprising given the small sample size and the fact that these samples are from two distinct trachoma populations. Though Western Europe was significantly different from all subgroups, the Russia and Americas subgroups were not significantly different by Fst. However, this could be attributed to the higher LGV representation in the Western European samples (15.5%) in comparison to the American samples (7.3%), and Russian samples (0%). Concordantly, when LGV samples were removed from the Western European dataset, Western Europe was no longer significantly different from Russia (**Table 5**). When evaluating only women without LGV from the Netherlands, Russia, and North America, the Russian women were not significantly different from the American and Netherlands women (Supplementary Table 8).

**Table 5:** Pairwise population differentiation (Fst) for regional subgroups.

	Africa	Asia	Americas	Russia	Western Europe	Western Europe non-LGV	Americas non-LGV
Africa		0.23421^∗^	0.27401^∗^	0.33802^∗^	0.17265^∗^		
Asia			0.36715^∗^	0.44728^∗^	0.29367^∗^		
Americas				0.01482 ns	0.02892^∗^		
Russia					0.05425^∗^	0.00305 ns	0.00884 ns
Western Europe							
Western Europe non-LGV							0.02627^∗^
Americas non-LGV							

^∗^*p < 0.05 by permutation test (*n* = 110)*.

### Phylogenetic Relationships and Evidence for Recombination

**FIGURE 1:**
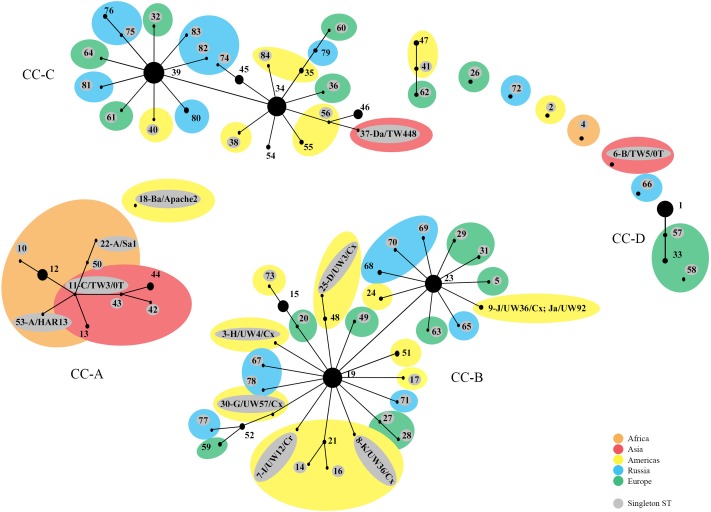
eBURST phylogeny for *Chlamydia trachomatis* geographic samples by ST. The numbers represent the Sequence Type (ST). STs that are linked differ at single multi-locus sequences (i.e., allelic number) and represent clonal groups. STs in the same clonal group are likely descended from the same recent ancestor, dependent on the region. Four distinct clonal clusters (CC) that correlate with disease phenotypes are shown: A, trachoma samples; B, non-prevalent urogenital samples; C, prevalent urogenital samples; and D, LGV samples. Large colored circles indicate what region multiple STs in a sub-group originated from. STs shaded gray are singleton STs. The area of the circle represents the number of samples of each ST. Orange, Africa; red, Asia; yellow, Americas; blue, Russia; green, Western Europe.

The phylogenetic relationships were initially evaluated by eBURST, which revealed clonal clusters (CC) similar to what we reported previously (**Figure 1**) (Dean et al., 2009; Batteiger et al., 2014) but with the addition of an LGV cluster. These included CC-A encompassing trachoma STs, CC-B with non-invasive, non-prevalent urogenital STs, CC-C with non-invasive prevalent STs and CC-D that included LGV STs. The predicted founders were ST19, ST23, ST34, and ST39 (Supplementary Figure 1 and Table 5). The 57 singleton STs are denoted by a gray circle alone or within a colored circle, representing a specific geographic region, but were not associated with any specific region.

**FIGURE 2:**
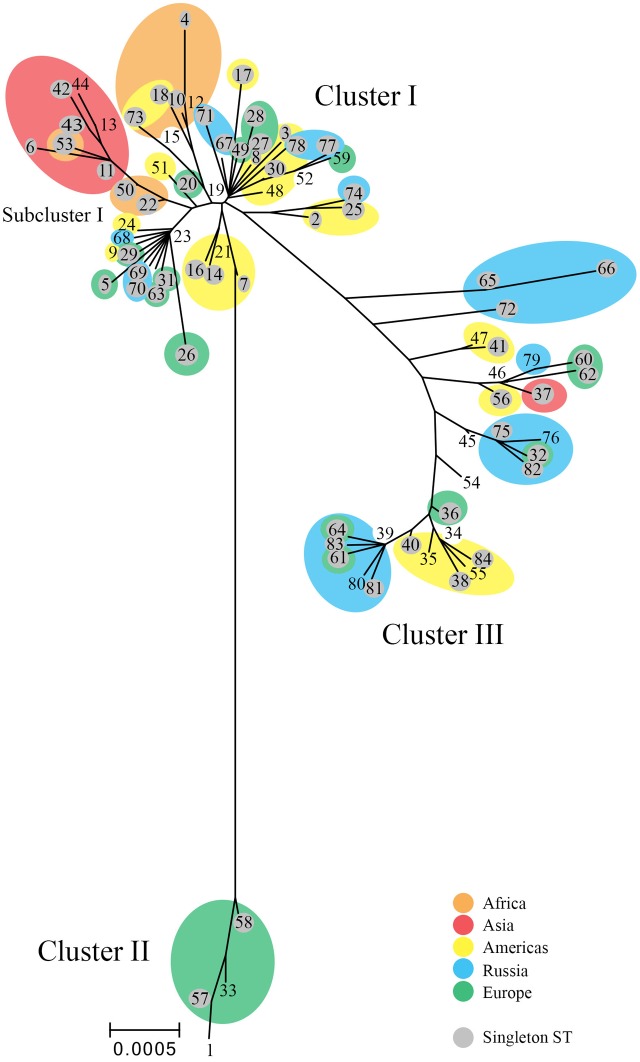
Phylogenetic tree for *C. trachomatis* nucleotide sequences by ST. The numbers represent the ST. The tree was constructed using the matrix of pairwise differences between the 84 ST concatenated nucleotide sequences for the seven MLST loci using the maximum composite likelihood method for estimating genetic distances. Only bootstrap values (1,000 replicates) of >70% are shown at branch points in the tree. STs (shaded gray) are singletons. Orange, Africa; red, Asia; yellow, Americas; blue, Russia; green, Western Europe. Scale bar indicates number of substitutions per site.

The tree (**Figure 2**) revealed ST branches similar to the eBURST clusters; both had branches or clusters for the disease phenotypic groups of LGV, non-invasive prevalent urogenital, and non-invasive non-prevalent urogenital STs. However, the trachoma STs formed a subgroup of the non-invasive non-prevalent urogenital branch. In addition, within disease groups, STs branched from central nodes by geographic region. These nodes contained, in general, large numbers of STs from diverse locations. For example, the founders ST19, ST23, ST34, and ST39 located at nodes in the tree contain STs from Europe, Russia, and the Americas. The amino acid tree showed a similar phylogeny (Supplementary Figure 1).

**FIGURE 3:**
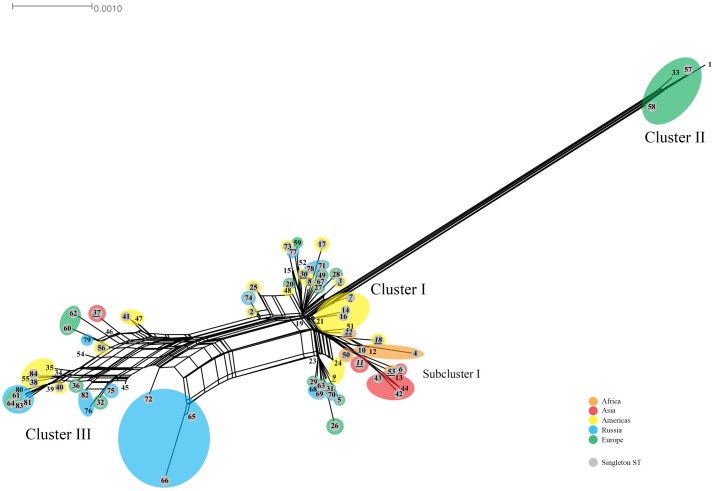
Splitstree for *C. trachomatis* nucleotide sequences by ST. The numbers represent the ST. The tree was obtained by using the seven loci for the 84 ST concatenated nucleotide sequences. STs (shaded gray) are singletons. Orange, Africa; red, Asia; yellow, Americas; blue, Russia; green, Western Europe. Scale bar indicates number of substitutions per site.

The Splitstree decomposition tree revealed evidence for a network structure consistent with homologous recombination (**Figure 3**). This was demonstrated by the interconnecting networks specifically among the ST founders ST19, ST23, ST34, and ST39 and other STs on the network in addition to the canonical evolutionary pathway shown in the tree (**Figure 2**). Supplementary Figure 2 shows the amino acid tree.

The Splitstree data are consistent with findings in the MLST and *omp*A genotyping data. Samples were classified as recombinant when the sequences of the seven genes that determine the ST were non-concordant with each other or with the seven gene sequences of the *Ct* genotype associated with the *omp*A genotype (**Table 6**), denoted in bold in Supplementary Table 3. There was no evidence of any recombination among the seven ST genes for any sample, although this would conceivably be possible.

**Table 6:** *Chlamydia trachomatis* recombinant samples in the dataset.

ST	Number of samples for ST	*omp*A genotype that matches ST sequences	Additional *omp*A genotypes for ST	Number of recombinants (%)^∗^	Geographic region
2	1	D	None	1 (100)	Americas
3	1	H	None	1 (100)	Americas
8	1	K	None	1 (100)	Americas
12	12	A	B, Ba	6 (50)	Tanzania
15	12	K	J	5 (42)	Americas, Russia
16	1	J	None	1 (100)	Americas
17	1	E	None	1 (100)	Americas
19	40	G	B, D, E, H, I, J, K	25 (62.5)	Americas, Europe, Russia
20	1	D	None	1 (100)	Europe
23	32	Ia	A, B, D, G, H, I, J, K	17 (53)	Americas, Europe, Russia
26	1	G	None	1 (100)	Europe
34	38	F	D, E, J, Ja	17 (45)	Americas, Europe
37	1	Da	None	1 (100)	Asia
39	45	E	D, G	2 (4.4)	Americas, Russia
41	1	Ja	None	1 (100)	Americas
45	8	F	D, E	5 (62.5)	Americas, Europe, Russia
46	9	E	D	1 (11)	Europe
49	1	K	None	1 (100)	Europe
50	1	B	None	1 (100)	Africa
53	1	A	None	1 (100)	Africa
60	1	D	None	1 (100)	Europe
62	1	D	None	1 (100)	Europe
65	1	K	None	1 (100)	Russia
66	1	B	None	1 (100)	Russia
68	2	B and G	B and G	2 (100)	Russia
69	1	J	None	1 (100)	Russia
71	1	E	None	1 (100)	Russia
72	1	D	None	1 (100)	Russia
73	1	J	None	1 (100)	Americas
74	1	G	None	1 (100)	Russia
78	1	D	None	1 (100)	Russia
79	2	D and Da	D and Da	2 (100)	Russia
84	1	K	None	1 (100)	Americas

^∗^*106 (32.8%) samples were recombinants among the entire database of 323*.

A total of 106 (32.8%) samples were considered putative recombinants. Excluding Asia and Africa where the sample sizes were small, Europe had a significantly higher number of recombinants than Russia and the Americas (*p* < 0.04) (**Table 2**), where, of the 109 samples, 49 were recombinant with 43 (87.8%) from the Netherlands. While *omp*A genotypes were generally consistent across samples of the same ST, many cases of recombinant strains were observed. For example, ST19 (*n* = 40) was primarily associated with *omp*A genotype G (37.5%) but eight different *omp*A genotypes (B, D, E, G, H, I, J, K) were also associated with this ST. The majority of these samples were from St. Petersburg and Amsterdam (Supplementary Table 3). ST23 (*n* = 32) was also associated with eight *omp*A genotypes (B, D, G, H, I, Ia, J, K) but the most frequent was Ia (47%). In contrast, the most geographically prevalent ST was ST39 (*n* = 45) where 95% were associated with *omp*A genotypes E. There were also 24 singletons that were recombinants (**Table 6**).

**Table 7** shows the *omp*A genotype, ST and allelic SNPs, if present, associated with each of the eight Boston samples added to the dataset. Under the column denoted ST sequence homology are the *Ct* *omp*A genotype sequences to which the sequences of the seven MLST genes are identical. For example, sample J/259b has four SNPs that are different from the sequences of the seven genes for reference and clinical J strains in the database. These SNPs are identical to the sequences of strains G and K, which suggests that this sample is a recombinant between J and G or K strains. Another example is sample D/256b; the *omp*A genotype is D but the MLST sequences of the seven genes are identical to reference and clinical F strains in the database. Therefore, it is a recombinant between D and F. Five (62.5%) of the eight Boston samples were putative recombinants. Supplementary Table 5 shows similar data for the 60 new samples from Amsterdam. For the Amsterdam samples, 43 (54.4%) of 79 samples were recombinants. Likewise, Supplementary Table 6 represents the data from St. Petersburg where 20 (34.5%) of 58 samples were recombinants.

**Table 7:** Evidence for recombination among the Boston, MA, United States *C. trachomatis* samples.

Sample	*omp*A genotype	ST sequence homology^∗^	ST	Location of allelic SNPs	
**J/253b**	**J**	**G or K**	**15 (G/K)**	**G: *pdhA*: 339 C→T *lysS*: 34 A→G**	**K: *pykF*: 40 G→A *lysS*: 34 A→G**
Ia/258b	Ia	Ia	23 (Ia)		
F/255b	F	F	34 (F)		
**D/256b**	**D**	**F**	**34 (F)**		
**D/257b**	**D**	**E**	**39 (E)**		
E/260b	E	E	39 (E)		
**J/259b**	**J**	**G or K**	**73 (G/K)**	**G: *pdhA*: 339 C→T *yhbG*: 433 G→C *lysS*: 34 A→G *leuS*: 282 T→C**	**K: *yhbG*: 433 G→C *pykF*: 40 G→A *lysS*: 34 A→G *leuS*: 282 T→C**
**K/254b**	**K**	**F**	**84 (F)**		

*Bold denotes putative recombinant. ^∗^The seven ST genes of the sample have the highest homology to the seven genes of the strain that has the *omp*A genotype denoted in the column (e.g., for sample D/256b that has a D *omp*A genotype, the seven ST genes were identical to the seven genes of F strains in the database where the *omp*A genotype was also F for those strains)*.

### Disease Phenotypes Are Associated with Specific Single Nucleotide Polymorphisms (SNPs)

**Table 8** shows the SNPs that are specific for each Haplotype and disease phenotype group. These groups include invasive urogenital disease caused by LGV strains (Haplotype 1), non-invasive urogenital disease group that includes the most prevalent worldwide strains including D, Da, E, and F (Haplotype 2), and trachoma caused by ocular A, B, Ba, and C strains (Haplotype 3). The non-invasive group includes clinical Ja strains that are recombinants of E and F strains. There are 14 SNPs that are specific for the LGV disease group, and each SNP independently identifies this group and Haplotype. The same is true for the non-invasive disease group. However, the two SNPs for the trachoma group together are specific for this group and Haplotype 3. Based on the Classification Index, the SNPs were not uniformly distributed.

**Table 8:** Single nucleotide polymorphisms (SNP) correlate with haplotype and disease phenotype.

Gene Locus	Number of SNPs per locus	Haplotype 1 All clinical and reference invasive LGV strains	Haplotype 2 Prevalent reference and clinical Da, E, F, and Ja strains (except clinical D/83s, D84s, D2s, D/EC, D/LC, D/SotonD5, D/SotonD6, D43nl, D/202nl, D/203nl, D/204nl, D/205nl, D/206nl, D/207nl, E87e, and reference D/UW3/Cx)	Haplotype 3 All reference and clinical trachoma A, B, Ba, and C strains (except reference strain A/Sa-1 and strain B/Jali20)
		Disease phenotype: LGV^∗^	Disease phenotype: urogenital non-invasive^∗^	Disease phenotype: trachoma^∗^
*glyA*	1–7			
*fmdhC*	1–5			
*pdhA*	1–6			
*yhbG*	1–21	SNPs 2–6	SNPs 15	
		8–12	18	
		16	20	
*pykF*	1–9		SNPs 6	SNP 3
			7	
*lysS*	1–11			
*leuS*	1–12	SNPs 1		SNP 3
		5		
		11		
**Total**	**71**	**14**	**5**	**2**

*LGV, lymphogranuloma venereum. ^∗^*p* < 0.01*.

## Discussion

The evolution of straining-typing techniques for bacteria have progressed from identifying variations in gel electrophoresis patterns and melt curve analyses to sequencing single pathogen-specific genes and MLST. While typing based on WGS would be ideal, this remains out of reach given the current expense and lack, in general, of sufficient DNA from clinical samples. However, it should be mentioned that we and others have been developing techniques to enrich DNA recovery directly from urogenital and ocular patient sample types with some success (Seth-Smith et al., 2013; Joseph et al., 2014; Hadfield et al., 2017).

*Omp*A genotyping remains widely used among *Chlamydia* investigators for molecular epidemiologic and comparative studies of strains between STD and trachoma populations. However, *omp*A encodes for the MOMP, which is under immune selection. MLST offers a more robust typing scheme by employing 6–8 housekeeping genes as relatively immutable signatures for strain typing (Maiden et al., 1998; Dean et al., 2009) and has become an important tool for studying both the epidemiology and evolution of human pathogens (Urwin and Maiden, 2003), including *Ct*.

In this study, we included 58 samples from Russia and eight from Boston, regions that have not previously been represented in *Ct* typing schemes. The 323 reference and clinical samples resolved into 84 STs, representing a 3.23 higher typing resolution over *omp*A genotyping consistent with previous studies (Dean et al., 2009; Gravningen et al., 2012; Batteiger et al., 2014; Herrmann et al., 2015). The high discriminatory index *D* of 0.98 (95% CI 0.97–0.99) and narrow CI for our MLST scheme confirms the validity of this typing method.

We noted an overall high rate of novel STs (67.9%), which may be expected because entire populations were not sampled and the numbers are small for some areas such as Asia and Africa. For example, there were 109 samples from Europe, representing six different countries, and 19 (68%) of the 28 STs were novel. Our findings are similar to other studies in Europe where the rates for novel STs were 62% among high school students in Norway (Gravningen et al., 2012), 65% among Tunisian sex workers (Gharsallah et al., 2016) and 62% among young adults in Amsterdam (Versteeg et al., 2015). Additional novel STs are likely to be identified as new regions undergo MLST. Indeed in Russia, of the 26 STs that contained Russian samples, 18 (69%) were novel.

A significantly higher ST to sample ratio of 0.45 was identified for Russian compared to European and American samples (*p* < 0.009). This was not explained by the number of STs unique to a region as the percentages were relatively uniform across the geographies (**Table 2**). However, Russian women had the highest nucleotide diversity (Pi = 0.00216), followed by North American women (Pi = 0.00210), and Dutch women (Pi = 0.00196), excluding LGV strains that are present only in the female Dutch population and would therefore skew the data. There are no males in the Russian dataset and therefore only women were evaluated here for the three locals. However, when assessing population differentiation for women from the Netherlands, Russia, and North America, excluding those with LGV, there were no significant differences (Supplementary Table 8). A larger sample size for each group would likely provide better resolution of the data. The increased sample ratio for Russia may reflect sexual mixing among the Russian STD cohort with the introduction of STs from other regions or reassortment of existing alleles. Of the 30 alleles present in Russia, 23 (76.7%) were found in other geographic regions, supporting the geographic influx of STs. Reassortment of alleles that generate new STs is also possible given the higher frequency of novel alleles in Russia at 43.3%. It has been shown that recombinational replacements are the major contributors to clonal diversity in contrast to point mutations among bacteria (Spratt et al., 2001). In support of our hypothesis, Hadfield et al. (2017) has shown that *Ct* evolves within genomic ‘ecotypes’ but also outside of these niches via recombination, consistent with prior genomic studies (Harris et al., 2012; Joseph et al., 2012). However, without a larger sample size from St. Petersburg, the relative overall diversity of STs will remain unknown, and we can only speculate as to the degree of reassortment based on the alleles comprising the STs that represent only the currently sampled cohort of women in Russia.

Excluding singleton STs, STs 23 and 34 were significantly associated with female STD patients in Amsterdam. We had previously noted that *omp*A genotyping is valuable as a separate adjunctive typing method along with MLST as it allows comparison with strains typed only by *omp*A and also can provide evidence for transmission and treatment efficacy as well as putative recombination (Batteiger et al., 2014). But *omp*A should not be included as one of the genes in the MLST scheme because it is under immune selection (Maiden et al., 1998; Dean et al., 2009). We performed *omp*A genotyping on all samples in the database and, for ST23, the samples comprised Ia, B, D, G, H, I, J, and K genotypes while for ST34 they included genotypes F, D, D2, E, J, and Ja (Supplementary Table 3). The high number of *omp*A genotypes for these two STs suggests a high rate of recombination. For example, the sequences of the seven MLST genes matched those of reference strain Ia/UW202 for 47% of the ST23 samples where the *omp*A genotype was also Ia. However, the remaining samples that also matched the seven MLST Ia/UW202 sequences had B, D, G, H, I, J, and K *omp*A genotypes, indicating a mismatch between the MLST and *omp*A sequences, providing evidence for recombination (Supplementary Table 3). Similarly, STs such as 15, 19, and 34 had numerous samples, some of which were putative recombinants. These findings are supported by partial and WGSs where *omp*A has been shown to be involved in frequent exchange and is considered a hotspot for recombination (Gomes et al., 2007; Harris et al., 2012; Joseph et al., 2012). For example, phylogenetic analyses indicate clustering of *omp*A Ja genotypes, which are uncommon, with highly prevalent *omp*A, D, E, and F genotypes (similar to ST34) where hotspots of recombination were noted in *omp*A and *pmp*EFGH genomic regions (Gomes et al., 2007; Joseph et al., 2012). Clusters of *omp*A D genotypes with less prevalent strains G, Ia, and J, similar to our strains in ST23, have also been noted (Harris et al., 2012). In a recent study, *omp*A Ba and C trachoma strains isolated from Australian Aborigines were found to cluster with urogenital D, Da, E, and F strains with hotspots also involving *omp*A and *pmp*EFGH (Andersson et al., 2016). Of course, with WGS, many additional genes have been found to be involved in recombination (Harris et al., 2012; Joseph et al., 2012; Hadfield et al., 2017).

To confirm putative recombinants, we had described that the sequences of the seven MLST genes were individually aligned to the respective gene for all samples in the database and compared these results to the *omp*A genotype of the same sample. For example, **Table 7** shows the five putative recombinants among the eight Boston samples. Sample D/256b had an *omp*A genotype of D but the seven MLST sequences were an exact match to the seven sequences of F. In some cases, one or more of the seven genes matched two different strains as was the case for samples J/253b and J/259b. Other examples of recombinants are shown in Supplementary Tables 6 and 7 for the Dutch and Russian samples, respectively. In addition, Splitstree analysis was performed and revealed ancillary evidence for a network structure consistent with homologous recombination (**Figure 2**). While these data confirm the recombinant nature of the samples, it is likely that there are other genetic regions that have undergone recombination and, therefore, it is not possible to determine the extent of genetic exchange unless WGS is performed (Joseph et al., 2011, 2012; Harris et al., 2012; Hadfield et al., 2017).

Overall, there were 106 (32.8%) putative recombinants (**Table 2** and Supplementary Table 3), which is similar to our previous studies and those of other investigators (Dean et al., 2009; Gravningen et al., 2012; Batteiger et al., 2014). Each geographic region contained recombinants, although Europe had a significantly higher number than Russia and the Americas (*p* < 0.04) (**Table 2**). This result was skewed by the higher rate of recombinants among the Amsterdam population, which would be expected given that the samples came from individuals at high risk for STDs where sexual mixing and import of strains from tourists could increase the chances for multiple *Ct* strain infections and opportunities for recombination. Indeed, rates of *Ct* mixed infections as high as 6 to 16% have been reported among men who have sex with men and heterosexual populations, respectively, in Europe, including the Netherlands (Quint et al., 2011; Rodriguez-Dominguez et al., 2015).

The most geographically prevalent ST was 39 with 45 samples, 95% of which had an *omp*A genotype of E; there were only two recombinants in this ST: one from Boston with a D *omp*A genotype and one from St. Petersburg with a G genotype. E genotypes are known to be the most globally prevalent (Lysén et al., 2004; Millman et al., 2004; Spaargaren et al., 2004; Lee et al., 2006; Gharsallah et al., 2016) and the least recombinogenic based on whole genome sequencing (Joseph et al., 2011, 2012). In our dataset, there were 72 E genotypes with an ST to sample ratio of 0.25; only the F genotype, the 4th most prevalent genotype with 30 samples, had a lower ratio at 0.17. The lower ratios indicate greater fitness as these strains are prevalent worldwide and have fewer allelic variants that resolve into fewer STs. This is borne out by the fact that only eight (11%) of the 72 E genotypes and 0 of the 30 F genotypes were recombinants (Supplementary Table 3). A recent genome study that included 149 E strains supports our conclusions (Hadfield et al., 2017). Genotype D was also highly prevalent but had a much higher ratio at 0.48, and 29 (88%) of 33 samples were recombinants.

We previously found that phylogeny based on MLST resolved the STs along disease phenotype demarcations. As samples have been added to the database, the phenotype resolution has increased to include the LGV phenotype, denoted as clonal cluster-D (CC-D; **Figure 1**). Similarly, the tree shows the three main clusters with the trachoma STs as a Subcluster of Cluster I, which resembles those of other reports (Herrmann et al., 2015).

To determine whether the phenotypic groups could be more finely discriminated, we analyzed the database for SNPs that independently or together would identify a phenotype. As in our previous studies (Dean et al., 2009; Batteiger et al., 2014), specific SNPs correlated with LGV, non-invasive urogenital disease and trachoma. Haplotype 2, which included the non-invasive urogenital disease group required exclusion of strains that were recombinants, specifically D genotypes.

## Author Contributions

Conceived and designed experiments: DD and VS. Wrote the paper: VS, AV, and DD. Assisted with editing the paper: EFvE, MPN, LAS, HJCdV, and SAM. Performed experiments: EFvE, MPN, TM, and BS. Analyzed data: VS, AV, EFvE, MPN, TM, BS, RW, and DD. Contributed reagents/materials/analysis tools: VS, LAS, HJCdV, JD, CE, SAM, and DD.

## Conflict of Interest Statement

The authors declare that the research was conducted in the absence of any commercial or financial relationships that could be construed as a potential conflict of interest.

## Footnotes

1 https://pubmlst.org/chlamydiales/

2 http://eburst.mlst.net

3 www.splitstree.org

4 http://www.r-project.org
