# Supplementary material for: Chlamydia trachomatis Strain Types Have Diversified Regionally and Globally with Evidence for Recombination across Geographic Divides
Source: Front Microbiol. 2017 Nov 13;8:2195. doi: 10.3389/fmicb.2017.02195 (PMC5693916; doi:10.3389/fmicb.2017.02195)
Supplement: Supplementary file 1 [file Data_Sheet_1.pdf]

Supplemental Material

Chlamydia trachomatis strain types have diversified regionally and globally with evidence for recombination across geographic divides

Vitaly Smelov[1,2,3]†, Alison Vrbanac[4]†§, Eleanne F. van Ess[5]†, Marlies P. Noz[5], Raymond Wan[4], Carina Eklund[2], Tyler Morgan[4], Lydia A. Shrier[6], Blake Sanders[4], Joakim Dillner[2], Henry J. C. de Vries[7,8,9], Servaas A. Morre[5,10], and Deborah Dean[4,11,12*]

[1]International Agency for Research on Cancer, World Health Organization, Lyon, France, [2]Karolinska Institute, Stockholm, Sweden, [3]North-Western State Medical University named after I.I. Mechnikov, St. Petersburg, Russia, [4]UCSF Benioff Children's Hospital Oakland Research Institute, Oakland, CA, United States, [5]Laboratory of Immunogenetics, Department of Medical Microbiology and Infection Control, VU University Medical Center, Amsterdam, the Netherlands, [6]Department of Pediatrics, Boston Children's Hospital, Boston, Massachusetts, United States, [7]Center for Infection and Immunology Amsterdam (CINIMA), Academic Medical Center, 1105 AZ, Amsterdam, The Netherlands, [8]Department of Dermatology, Academic Medical Center, 1105 AZ, Amsterdam, The Netherlands, [9]STI Outpatient Clinic, Public Health Service of Amsterdam (GGD Amsterdam), 1018 WT, Amsterdam, The Netherlands, [10]Institute of Public Health Genomics, Department of Genetics and Cell Biology, Research Institute GROW (School for Oncology & Developmental Biology), Faculty of Health, Medicine & Life Sciences, University of Maastricht, Maastricht, The Netherlands, [11]Department of Bioengineering, University of California at Berkeley and University of California at San Francisco, CA, United States, [12]Departments of Medicine and Pediatrics, UCSF, San Francisco, CA, United States

Tables

Supplementary Table 1. Primers used for PCR and Sequencing.

Supplementary Table 2. Characteristics of Alleles for Each Locus.

Supplementary Table 3. Sequence types, allelic profiles, and clinical characteristics of reference and clinical samples.

Supplementary Table 4: eBURST report for 323 isolates.

Supplementary Table 5: STs by geographic region.

Supplemental Table 6: Evidence of recombination for samples from Amsterdam, The Netherlands.

Supplemental Table 7: Evidence of recombination for samples from St. Petersburg, Russia.

Supplementary Table 8: Pairwise Population Differentiation (Fst) for North American, Dutch, and Russian Women**.**

Figures

Supplementary Figure 1: Minimum evolution tree for *C. trachomatis* amino acid sequences by ST. The numbers represent the Sequence Type (ST). The tree was constructed using the matrix of pairwise differences between the 84 ST concatenated amino acid sequences for the seven loci using the maximum composite likelihood method for estimating genetic distances. Only bootstrap values (1,000 replicates) >70% are represented at branch points in the tree. STs shaded grey are singleton STs. The area of the circle represents the number of samples of each ST. Orange, Africa; red, Asia; yellow, The America; blue, Russia; green, Western Europe. Scale bar indicates number of substitutions per site.

Supplementary Figure 2: Splitstree for *C. trachomatis* amino acid sequences by ST. The numbers represent the Sequence Type (ST). The tree was obtained by using the 7 loci for the 84 ST concatenated sequences. STs shaded grey are singleton STs. The area of the circle represents the number of samples of each ST. Orange, Africa; red, Asia; yellow, The America; blue, Russia; green, Western Europe. Scale bar indicates number of substitutions per site.

Supplementary Table 1: Primers used for PCR and Sequencing

Target Locus	Region	PCR Primer Label	Sequence 5' to 3'	Sequencing Primer Label	Sequence 5' to 3' [2]	Length of Sequence, bp
glyA	CT432	1FglyA<br>1RglyA	GAAGACTGTGGCGCTGTT<br>CTTCCTGAGCGATCCCTTC	G1FSP<br>G1RSP	GCTCATTTCGCAGGCTTGGTT<br>GACAGCTCTCCCTCACTTT	523
		Alternates:<br>G1PCRFa<br>G1PCRRa	 GAACATAAGCCCACCGTTCT<br>TTCCAGATCGATTTCAGGAT	1FglyA<br>1RglyA	GAAGACTGTGGCGCTGTTT<br>CTTCCTGAGCGATCCCTTC	
mdhC	CT376	3FmdhC<br>3RmdhC	GGAGATGTTTTTGGCCTTG<br>CGATTACTGCACTACCACG	G3FSP<br>G3RSP	GATGTTTTTGGCCTTGATTG<br>CTGTACAGAAGGCACCATAATA	521
		Alternates:<br>G3PCRFa<br>G3PCRRa	 AGGGCAAATAGCCTATAGCT<br>AAGCTCGTGCTGCAGAAGCT	3FmdhC<br>3RmdhC	GGAGATGTTTTTGGCCTTG<br>CGATTACTGCACTACCACG	
pdhA	CT245	5FpdhA<br>5RpdhA	CTACAGAAGCCCGAGTTTT<br>CTGTTTGTTGCATGTGGTG	G5FSP<br>G5RSP	ATTCTTTCTGCATTGACCT<br>CATGTGGTGATAAGCTTCTCTAAA	551
		Alternate:<br>G5PCRFa<br>G5PCRRa	 CATCCTCTGACTCTCAACAT<br>TAGGATCGGAAATAGAGTGT	 5FpdhA<br>5RpdhA	 CTACAGAAGCCCGAGTTTT<br>CTGTTTGTTGCATGTGGTG	
ybhG	CT653	8FyhbG<br>8RybhG	TCAAGTCAATGCAGGAGAA<br>GATAGTGTTGACGTACCATAGGAT	G8FSP<br>G8RSP	CCTAACGGAGCGGGGAAA<br>GAGCAGACGATCCTTCAA	504
pykF	CT332	9FpykF<br>9RpykF	ATCTTATCGCTGCTTCGTT<br>CAGCAATAATAGGGAGATA	G9SeqF<br>G9SeqR	TGTAATGAAGATATTGACA<br>TTGGATAAAAACATCGGAG	527
		Alternates:<br>G9seqF<br>G9seqR	 TGTAATGAAGATATTGACAGCAT<br>TTGGATAAAAACATCGGAG	 G9SeqF2<br>G9SeqR2	 GATATTGACAGCATGCGTAAAGTT<br>ATTGTGTATACCCAGACGGGAGGAT	
lysS	CT781	11FPCR2<br>11RPCR2	GAATGTCCCGAGTTTATGAA<br>GTATAGAAGCAAAAAAAGAT	11FlysS<br>11RlysS	GAAGGAATCGATAGAACGCATAAT<br>ATACGCCGCATAACAGGGAAAAAC	577
		Alternates:<br>G11FSP<br>G11RSP	 TCCAGAGTTTACCATGATAGAGG<br>AACAGGGAAAAACAGGACAT	 11seqF<br>11seqR	 TATGCGGCTTATTGGGATTA<br>AATGGATGCGGCGTCTGTCAA	
leuS	CT209	12F5<br>12R5	GAGCTGGATTGGCCAGAAAGT<br>GCCAGAAACGCGCATAAAGT	12DeanF5<br>G12PCRRa<br>G12RSP	TCTTCAGGAGCTTCTGTTAATT<br>AGTGCAGTACAGCATGTTCT<br>AAGAGCCTGCCCATTGAG	521
		Alternates:<br>G12PCRFa<br>G12PCRRa	 ACAAGACCGGACACTTTGAT<br>AGTGCAGTACAGCATGTTCT	 G12PCRFa<br>G12RSP	 ACAAGACCGGACACTTTGAT<br>CTCAATGGGCAGGCTCTT	

Supplementary Table 2. Characteristics of alleles for each locus

Gene locus	No. alleles	Length, bp	No. polymorphic sites	Average pairwise distance	Average dS	Average dN
glyA	12	523	9	0.0032	0.0019	0.0013
mdhC	7	521	6	0.0013	0.0009	0.0004
pdhA	8	551	6	0.0002	0.0001	0.0001
yhbG	11	504	22	0.0109	0.0100	0.0009
pykF	10	527	13	0.0029	0.0013	0.0016
lysS	12	577	15	0.0015	0.0011	0.0004
leuS	18	521	19	0.0024	0.0007	0.0017
Total	78	3,724	90	0.0224	0.0160	0.0064

Supplementary Table 3. Sequence types, allelic profiles, and clinical characteristics of reference and clinical samples*

		Allele assignment for each locus								
Strain ID †	ST	*glyA*	*mdhC*	*pdhA*	*yhbG*	*pykF*	*lysS*	*leuS*	Region of isolation	Diagnosis/Site
L1/440	1	1	1	3	8	1	4	11	California	LGV
L2/434	1	“	“	“	“	“	“	“	California	LGV
L3/404	1	“	“	“	“	“	“	“	California	LGV
L2b/Canada1	1	“	“	“	“	“	“	“	Canada	Proctitis
L2b/Canada2	1	“	“	“	“	“	“	“	Canada	Proctitis
L2b/CV204	1	“	“	“	“	“	“	“	France	Proctitis
L2b/LST	1	“	“	“	“	“	“	“	France	Proctitis
L2b/795	1	“	“	“	“	“	“	“	France	Proctitis
L2/54s	1	“	“	“	“	“	“	“	San Francisco	Proctitis
L2a/UW396	1	“	“	“	“	“	“	“	Seattle	LGV
L1/115	1	“	“	“	“	“	“	“	South Africa	LGV
L1/1322/p2	1	“	“	“	“	“	“	“	South Africa	LGV
L1/224	1	“	“	“	“	“	“	“	South Africa	LGV
L2b/8200/07	1	“	“	“	“	“	“	“	Sweden	Proctitis
L2b/Ams1	1	“	“	“	“	“	“	“	The Netherlands	Proctitis
L2b/Ams2	1	“	“	“	“	“	“	“	The Netherlands	Proctitis
L2b/Ams3	1	“	“	“	“	“	“	“	The Netherlands	Proctitis
L2b/Ams4	1	“	“	“	“	“	“	“	The Netherlands	Proctitis
L2b/Ams5	1	“	“	“	“	“	“	“	The Netherlands	Proctitis
L2b/86nl	1	“	“	“	“	“	“	“	The Netherlands	Proctitis
L2/237nl	1	“	“	“	“	“	“	“	The Netherlands	Cervix
L2b/UCH1	1	“	“	“	“	“	“	“	United Kingdom	Proctitis
L2b/UCH2	1	“	“	“	“	“	“	“	United Kingdom	Proctitis
L2/25667R	1	“	“	“	“	“	“	“	USA	Proctitis
L2c	1	“	“	“	“	“	“	“	San Francisco	Proctitis
**D/84s**	**2**	**2**	**3**	**3**	**6**	**5**	**4**	**3**	**San Francisco**	**Cervicitis**
**H/UW4/Cx**	**3**	**3**	**1**	**3**	**6**	**6**	**4**	**3**	**Washington**	**Cervicitis**
A/51t	4	3	3	1	6	3	7	9	Tanzania	Trachoma
H/46nl	5	3	3	2	6	6	8	3	The Netherlands	Cervicitis and vaginal discharge
B/TW5/OT	6	3	3	3	4	3	5	10	Taiwan	Conjunctivitis
I/UW12/Ur	7	3	3	3	6	1	4	3	Washington	Urethritis
**K/UW36/Cx**	**8**	**3**	**3**	**3**	**6**	**2**	**4**	**3**	**Washington**	**Cervicitis**
J/UW36/Cx	9	3	3	3	6	2	8	3	Washington	Cervicitis
Ja/UW92	9	“	“	“	“	“	“	“	Washington	Cervicitis
B/53t	10	3	3	3	6	3	4	9	Tanzania	Trachoma
C/TW3/OT	11	3	3	3	6	3	5	7	Taiwan	Conjunctivitis
A/2497	12	3	3	3	6	3	5	9	Tanzania	Trachoma
A/363	12	“	“	“	“	“	“	“	Tanzania	Trachoma
A/48t	12	“	“	“	“	“	“	“	Tanzania	Trachoma
A/5291	12	“	“	“	“	“	“	“	Tanzania	Trachoma
A/59t	12	“	“	“	“	“	“	“	Tanzania	Trachoma
A/7249	12	“	“	“	“	“	“	“	Tanzania	Trachoma
**B/TZ1A828/OT**	**12**	**“**	**“**	**“**	**“**	**“**	**“**	**“**	**Tanzania**	**Trachoma**
**B/50t**	**12**	**“**	**“**	**“**	**“**	**“**	**“**	**“**	**Tanzania**	**Trachoma**
**B/60t**	**12**	**“**	**“**	**“**	**“**	**“**	**“**	**“**	**Tanzania**	**Trachoma**
**B/61t**	**12**	**“**	**“**	**“**	**“**	**“**	**“**	**“**	**Tanzania**	**Trachoma**
**B/62t**	**12**	**“**	**“**	**“**	**“**	**“**	**“**	**“**	**Tanzania**	**Trachoma**
**Ba/52t**	**12**	**“**	**“**	**“**	**“**	**“**	**“**	**“**	**Tanzania**	**Trachoma**
C/32n	13	3	3	3	6	3	6	7	Nepal	Trachoma, TS
C/33n	13	“	“	“	“	“	“	“	Nepal	Trachoma, TS
G/15s	14	3	3	3	6	4	4	8	San Francisco	Proctitis
K/186i	15	3	3	3	6	6	1	6	Indianapolis	Urethra
K/187i	15	“	“	“	“	“	“	“	Indianapolis	Cervix
K/296sp	15	“	“	“	“	“	“	“	St. Petersburg	Cervicitis
K/305sp	15	“	“	“	“	“	“	“	St. Petersburg	Cervicitis

K/42nl	15	“	“	“	“	“	“	“	The Netherlands	Cervicitis w/ vaginal discharge
K/49nl	15	“	“	“	“	“	“	“	The Netherlands	Cervicitis w/ vaginal discharge
K/250nl	15	“	“	“	“	“	“	“	The Netherlands	Cervix
**J/253b**	**15**	**“**	**“**	**“**	**“**	**“**	**“**	**“**	**Boston**	**Cervicitis**
**J/112i**	**15**	**“**	**“**	**“**	**“**	**“**	**“**	**“**	**Indianapolis**	**Urethra**
**J/113i**	**15**	**“**	**“**	**“**	**“**	**“**	**“**	**“**	**Indianapolis**	**Cervix**
**J/31-98**	**15**	**“**	**“**	**“**	**“**	**“**	**“**	**“**	**Seattle**	**Cervicitis**
**J/318sp**	**15**	**“**	**“**	**“**	**“**	**“**	**“**	**“**	**St. Petersburg**	**Cervicitis**
**J/27s**	**16**	**3**	**3**	**3**	**6**	**6**	**1**	**8**	**San Francisco**	**Cervicitis/urethritis**
**E/87e**	**17**	**3**	**3**	**3**	**6**	**6**	**2**	**3**	**Ecuador**	**Cervicitis**
Ba/Apache2	18	3	3	3	6	6	3	9	Arizona	Conjunctivitis
G/266sp	19	3	3	3	6	6	4	3	St. Petersburg	Cervicitis
G/271sp	19	“	“	“	“	“	“	“	St. Petersburg	Cervicitis
G/273sp	19	“	“	“	“	“	“	“	St. Petersburg	Cervicitis
G/284sp	19	“	“	“	“	“	“	“	St. Petersburg	Cervicitis
G/286sp	19	“	“	“	“	“	“	“	St. Petersburg	Cervicitis
G/295sp	19	“	“	“	“	“	“	“	St. Petersburg	Cervicitis
G/301sp	19	“	“	“	“	“	“	“	St. Petersburg	Cervicitis
G/303sp	19	“	“	“	“	“	“	“	St. Petersburg	Cervicitis
G/315sp	19	“	“	“	“	“	“	“	St. Petersburg	Cervicitis
G/317sp	19	“	“	“	“	“	“	“	St. Petersburg	Cervicitis
G/225nl	19	“	“	“	“	“	“	“	The Netherlands	Cervix
G/227nl	19	“	“	“	“	“	“	“	The Netherlands	Cervix
G/229nl	19	“	“	“	“	“	“	“	The Netherlands	Cervicitis
G/230nl	19	“	“	“	“	“	“	“	The Netherlands	PID
G/SotonG1	19	“	“	“	“	“	“	“	United Kingdom	Cervicitis
**B/193nl**	**19**	**“**	**“**	**“**	**“**	**“**	**“**	**“**	**The Netherlands**	**Cervicitis**
**B/194nl**	**19**	**“**	**“**	**“**	**“**	**“**	**“**	**“**	**The Netherlands**	**PID**
**D/83s**	**19**	**“**	**“**	**“**	**“**	**“**	**“**	**“**	**San Francisco**	**Cervicitis**
**D/202nl**	**19**	**“**	**“**	**“**	**“**	**“**	**“**	**“**	**The Netherlands**	**Cervix**
**D/203nl**	**19**	**“**	**“**	**“**	**“**	**“**	**“**	**“**	**The Netherlands**	**Cervix**
**D/204nl**	**19**	**“**	**“**	**“**	**“**	**“**	**“**	**“**	**The Netherlands**	**Cervix**
**D/205nl**	**19**	**“**	**“**	**“**	**“**	**“**	**“**	**“**	**The Netherlands**	**Cervicitis**
**D/206nl**	**19**	**“**	**“**	**“**	**“**	**“**	**“**	**“**	**The Netherlands**	**PID**
**D/207nl**	**19**	**“**	**“**	**“**	**“**	**“**	**“**	**“**	**The Netherlands**	**PID**
**D/SotonD5**	**19**	**“**	**“**	**“**	**“**	**“**	**“**	**“**	**United Kingdom**	**Cervicitis**
**D/SotonD6**	**19**	**“**	**“**	**“**	**“**	**“**	**“**	**“**	**United Kingdom**	**Cervicitis**
**E/279sp**	**19**	**“**	**“**	**“**	**“**	**“**	**“**	**“**	**St. Petersburg**	**Cervicitis**
**E/294sp**	**19**	**“**	**“**	**“**	**“**	**“**	**“**	**“**	**St. Petersburg**	**Cervicitis**
**H/114i**	**19**	**“**	**“**	**“**	**“**	**“**	**“**	**“**	**Indianapolis**	**Urethra**
**H/115i**	**19**	**“**	**“**	**“**	**“**	**“**	**“**	**“**	**Indianapolis**	**Cervix**
**H/18s**	**19**	**“**	**“**	**“**	**“**	**“**	**“**	**“**	**San Francisco**	**Cervicitis/urethritis**
**H/40nl**	**19**	**“**	**“**	**“**	**“**	**“**	**“**	**“**	**The Netherlands**	**Cervicitis**
**I/22p**	**19**	**“**	**“**	**“**	**“**	**“**	**“**	**“**	**Lisbon**	**Cervicitis/urethritis**
**I/240nl**	**19**	**“**	**“**	**“**	**“**	**“**	**“**	**“**	**The Netherlands**	**PID**
**I/241nl**	**19**	**“**	**“**	**“**	**“**	**“**	**“**	**“**	**The Netherlands**	**Cervix**
**I/245nl**	**19**	**“**	**“**	**“**	**“**	**“**	**“**	**“**	**The Netherlands**	**PID**
**J/44nl**	**19**	**“**	**“**	**“**	**“**	**“**	**“**	**“**	**The Netherlands**	**Cervicitis**
**K/267sp**	**19**	**“**	**“**	**“**	**“**	**“**	**“**	**“**	**St. Petersburg**	**Cervicitis**
**K/248nl**	**19**	**“**	**“**	**“**	**“**	**“**	**“**	**“**	**The Netherlands**	**Cervix**
**K/249nl**	**19**	**“**	**“**	**“**	**“**	**“**	**“**	**“**	**The Netherlands**	**Cervix**
**D/43nl**	**20**	**3**	**3**	**3**	**6**	**6**	**4**	**6**	**The Netherlands**	**Cervicitis and vaginal discharge**
G/13s	21	3	3	3	6	6	4	8	San Francisco	Proctitis
G/14s	21	“	“	“	“	“	“	“	San Francisco	Proctitis
A/Sa1	22	3	3	3	6	6	5	2	Saudi Arabia	Trachoma
Ia/57e	23	3	3	3	6	6	8	3	Ecuador	Cervicitis
Ia/94i	23	“	“	“	“	“	“	“	Indianapolis	Urethra

Ia/95i	23	“	“	“	“	“	“	“	Indianapolis	Cervix
Ia/118i	23	“	“	“	“	“	“	“	Indianapolis	Cervix
Ia/119i	23	“	“	“	“	“	“	“	Indianapolis	Urethra
Ia4/177i	23	“	“	“	“	“	“	“	Indianapolis	Urethra
Ia4/180i	23	“	“	“	“	“	“	“	Indianapolis	Cervix
Ia/178i	23	“	“	“	“	“	“	“	Indianapolis	Cervix
Ia/179i	23	“	“	“	“	“	“	“	Indianapolis	Urethra
Ia/183i	23	“	“	“	“	“	“	“	Indianapolis	Urethra
Ia/184i	23	“	“	“	“	“	“	“	Indianapolis	Cervix
Ia/SotonIa1	23	“	“	“	“	“	“	“	United Kingdom	Cervicitis
Ia/SotonIa3	23	“	“	“	“	“	“	“	United Kingdom	Cervicitis
Ia/UW202	23	“	“	“	“	“	“	“	Washington	Cervicitis
Ia/258b	23	“	“	“	“	“	“	“	Boston	
**B/298sp**	**23**	**“**	**“**	**“**	**“**	**“**	**“**	**“**	**St. Petersburg**	**Cervicitis**
**D/2s**	**23**	**“**	**“**	**“**	**“**	**“**	**“**	**“**	**San Francisco**	**Cervicitis/urethritis**
**G/268sp**	**23**	**“**	**“**	**“**	**“**	**“**	**“**	**“**	**St. Petersburg**	**Cervicitis**
**H/231nl**	**23**	**“**	**“**	**“**	**“**	**“**	**“**	**“**	**The Netherlands**	**Cervix**
**H/232nl**	**23**	**“**	**“**	**“**	**“**	**“**	**“**	**“**	**The Netherlands**	**Cervix**
**H/233nl**	**23**	**“**	**“**	**“**	**“**	**“**	**“**	**“**	**The Netherlands**	**Cervicitis**
**H/234nl**	**23**	**“**	**“**	**“**	**“**	**“**	**“**	**“**	**The Netherlands**	**PID**
**I/235nl**	**23**	**“**	**“**	**“**	**“**	**“**	**“**	**“**	**The Netherlands**	**Cervix**
**I/236nl**	**23**	**“**	**“**	**“**	**“**	**“**	**“**	**“**	**The Netherlands**	**Cervix**
**I/238nl**	**23**	**“**	**“**	**“**	**“**	**“**	**“**	**“**	**The Netherlands**	**Cervicitis**
**I/239nl**	**23**	**“**	**“**	**“**	**“**	**“**	**“**	**“**	**The Netherlands**	**Cervicitis**
**I/242nl**	**23**	**“**	**“**	**“**	**“**	**“**	**“**	**“**	**The Netherlands**	**Cervix**
**I/243nl**	**23**	**“**	**“**	**“**	**“**	**“**	**“**	**“**	**The Netherlands**	**Cervicitis**
**I/244nl**	**23**	**“**	**“**	**“**	**“**	**“**	**“**	**“**	**The Netherlands**	**Cervicitis**
**J/311sp**	**23**	**“**	**“**	**“**	**“**	**“**	**“**	**“**	**St. Petersburg**	**Cervicitis**
**J/247nl**	**23**	**“**	**“**	**“**	**“**	**“**	**“**	**“**	**The Netherlands**	**Cervix**
**K/251nl**	**23**	**“**	**“**	**“**	**“**	**“**	**“**	**“**	**The Netherlands**	**Cervicitis**
Ia/24s	24	3	3	3	6	6	8	8	San Francisco	Cervicitis/urethritis
Ia/25s	24	“	“	“	“	“	“	“	San Francisco	Cervicitis/urethritis
D/UW3/Cx	25	3	3	3	6	7	4	1	Washington	Cervicitis
**G/16p**	**26**	**3**	**3**	**3**	**7**	**6**	**8**	**5**	**Lisbon**	**Cervicitis/urethritis**
G/17p	27	3	3	4	6	6	4	3	Lisbon	Cervicitis/urethritis
Ia/23p	28	3	3	4	6	6	4	4	Lisbon	Cervicitis/urethritis
H/21p	29	3	3	4	6	6	8	3	Lisbon	Cervicitis
G/UW57/Cx	30	3	3	5	6	6	4	3	Washington	Cervicitis
H/20p	31	3	3	7	6	6	8	3	Lisbon	Cervicitis/urethritis
F/38nl	32	4	4	3	2	7	4	3	The Netherlands	Cervicitis and vaginal discharge
L2b/48nl	33	5	2	3	8	1	4	11	The Netherlands	Proctitis
L2b/85nl	33	“	“	“	“	“	“	“	The Netherlands	Proctitis
F/ICCal3	34	6	3	3	2	7	4	3	Boston	
F/98i	34	“	“	“	“	“	“	“	Indianapolis	Urethra
F/99i	34	“	“	“	“	“	“	“	Indianapolis	Cervix
F/181i	34	“	“	“	“	“	“	“	Indianapolis	Urethra
F/182i	34	“	“	“	“	“	“	“	Indianapolis	Cervix
F/191i	34	“	“	“	“	“	“	“	Indianapolis	Urethra
F/192i	34	“	“	“	“	“	“	“	Indianapolis	Cervix
F/8p	34	“	“	“	“	“	“	“	Lisbon	Cervicitis/urethritis
F/9p	34	“	“	“	“	“	“	“	Lisbon	Cervicitis/urethritis
F/289sp	34	“	“	“	“	“	“	“	St. Petersburg	cervicitis
F/312sp	34	“	“	“	“	“	“	“	St. Petersburg	cervicitis
F/313sp	34	“	“	“	“	“	“	“	St. Petersburg	cervicitis
F/SW4	34	“	“	“	“	“	“	“	Sweden	Cervicitis
F/SW5	34	“	“	“	“	“	“	“	Sweden	Cervicitis
F/217nl	34	“	“	“	“	“	“	“	The Netherlands	Cervix
F/218nl	34	“	“	“	“	“	“	“	The Netherlands	Cervix
F/219nl	34	“	“	“	“	“	“	“	The Netherlands	Cervix

F/220nl	34	“	“	“	“	“	“	“	The Netherlands	Cervicitis
F/221nl	34	“	“	“	“	“	“	“	The Netherlands	PID
F/SotonF3	34	“	“	“	“	“	“	“	United Kingdom	Cervicitis
F/255b	34	“	“	“	“	“	“	“	Boston	
**D/256b**	**34**	**“**	**“**	**“**	**“**	**“**	**“**	**“**	**Boston**	**Cervicitis**
**D2/96i**	**34**	**“**	**“**	**“**	**“**	**“**	**“**	**“**	**Indianapolis**	**Urethra**
**D2/97i**	**34**	**“**	**“**	**“**	**“**	**“**	**“**	**“**	**Indianapolis**	**Cervix**
**D2/189i**	**34**	**“**	**“**	**“**	**“**	**“**	**“**	**“**	**Indianapolis**	**Cervix**
**D2/190i**	**34**	**“**	**“**	**“**	**“**	**“**	**“**	**“**	**Indianapolis**	**Urethra**
**D/196nl**	**34**	**“**	**“**	**“**	**“**	**“**	**“**	**“**	**The Netherlands**	**Cervix**
**D/197nl**	**34**	**“**	**“**	**“**	**“**	**“**	**“**	**“**	**The Netherlands**	**Cervix**
**D/200nl**	**34**	**“**	**“**	**“**	**“**	**“**	**“**	**“**	**The Netherlands**	**Cervicitis**
**D/201nl**	**34**	**“**	**“**	**“**	**“**	**“**	**“**	**“**	**The Netherlands**	**PID**
**D/209nl**	**34**	**“**	**“**	**“**	**“**	**“**	**“**	**“**	**The Netherlands**	**Cervix**
**D/SotonD1**	**34**	**“**	**“**	**“**	**“**	**“**	**“**	**“**	**United Kingdom**	**Cervicitis**
**E/19e**	**34**	**“**	**“**	**“**	**“**	**“**	**“**	**“**	**Ecuador**	**Cervicitis**
**E/5s**	**34**	**“**	**“**	**“**	**“**	**“**	**“**	**“**	**San Francisco**	**Cervicitis/urethritis**
**E/214nl**	**34**	**“**	**“**	**“**	**“**	**“**	**“**	**“**	**The Netherlands**	**PID**
**Ja/41nl**	**34**	**“**	**“**	**“**	**“**	**“**	**“**	**“**	**The Netherlands**	**Cervicitis and vaginal discharge**
**Ja/47nl**	**34**	**“**	**“**	**“**	**“**	**“**	**“**	**“**	**The Netherlands**	**Cervicitis and vaginal discharge**
**J/252nl**	**34**	**“**	**“**	**“**	**“**	**“**	**“**	**“**	**The Netherlands**	**PID**
F/10s	35	6	3	3	2	7	4	8	San Francisco	PID
F/11s	35	“	“	“	“	“	“	“	San Francisco	PID
F/12s	35	“	“	“	“	“	“	“	San Francisco	PID
E/39nl	36	6	3	3	3	7	4	3	The Netherlands	Cervicitis
**Da/TW448**	**37**	**6**	**3**	**3**	**5**	**7**	**4**	**2**	**Taiwan**	**Trachoma**
D/3s	38	6	3	6	2	7	4	3	San Francisco	Cervicitis/urethritis
E/Bour	39	6	4	3	2	7	4	3	California	Cervicitis
E/28e	39	“	“	“	“	“	“	“	Ecuador	Cervicitis
E/55e	39	“	“	“	“	“	“	“	Ecuador	Cervicitis
E/56e	39	“	“	“	“	“	“	“	Ecuador	Cervicitis
E/88i	39	“	“	“	“	“	“	“	Indianapolis	Cervix
E/89i	39	“	“	“	“	“	“	“	Indianapolis	Urethra
E/102i	39	“	“	“	“	“	“	“	Indianapolis	Urethra
E/103i	39	“	“	“	“	“	“	“	Indianapolis	Cervix
E/106i	39	“	“	“	“	“	“	“	Indianapolis	Urethra
E/107i	39	“	“	“	“	“	“	“	Indianapolis	Cervix
E/108i	39	“	“	“	“	“	“	“	Indianapolis	Cervix
E/109i	39	“	“	“	“	“	“	“	Indianapolis	Urethra
E/110i	39	“	“	“	“	“	“	“	Indianapolis	Cervix
E/111i	39	“	“	“	“	“	“	“	Indianapolis	Urethra
E/116i	39	“	“	“	“	“	“	“	Indianapolis	Urethra
E/117i	39	“	“	“	“	“	“	“	Indianapolis	Urethra (female)
E6/120i	39	“	“	“	“	“	“	“	Indianapolis	Cervix
E6/121i	39	“	“	“	“	“	“	“	Indianapolis	Urethra
E/171i	39	“	“	“	“	“	“	“	Indianapolis	Urethra
E/172i	39	“	“	“	“	“	“	“	Indianapolis	Cervix
E/6p	39	“	“	“	“	“	“	“	Lisbon	Cervicitis
E/7p	39	“	“	“	“	“	“	“	Lisbon	Cervicitis
E/150	39	“	“	“	“	“	“	“	Seattle	Proctitis
E/11023	39	“	“	“	“	“	“	“	Seattle	Cervicitis
E/260b	39	“	“	“	“	“	“	“	Boston	
E/275sp	39	“	“	“	“	“	“	“	St. Petersburg	cervicitis
E/276sp	39	“	“	“	“	“	“	“	St. Petersburg	cervicitis
E/278sp	39	“	“	“	“	“	“	“	St. Petersburg	cervicitis
E/280sp	39	“	“	“	“	“	“	“	St. Petersburg	cervicitis
E/285sp	39	“	“	“	“	“	“	“	St. Petersburg	cervicitis
E/290sp	39	“	“	“	“	“	“	“	St. Petersburg	cervicitis

E/292sp	39	“	“	“	“	“	“	“	St. Petersburg	cervicitis
E/299sp	39	“	“	“	“	“	“	“	St. Petersburg	cervicitis
E/307sp	39	“	“	“	“	“	“	“	St. Petersburg	cervicitis
E/SW2	39	“	“	“	“	“	“	“	Sweden	Urethritis
E/SW3	39	“	“	“	“	“	“	“	Sweden	Cervicitis
E/58t	39	“	“	“	“	“	“	“	Tanzania	Conjunctivitis
E/45nl	39	“	“	“	“	“	“	“	The Netherlands	Cervicitis
E/213nl	39	“	“	“	“	“	“	“	The Netherlands	cervicitis
E/215nl	39	“	“	“	“	“	“	“	The Netherlands	PID
E/216nl	39	“	“	“	“	“	“	“	The Netherlands	PID
E/SotonE4	39	“	“	“	“	“	“	“	United Kingdom	Cervicitis
E/SotonE8	39	“	“	“	“	“	“	“	United Kingdom	Cervicitis
**D/257b**	**39**	**“**	**“**	**“**	**“**	**“**	**“**	**“**	**Boston**	**Cervicitis**
**G/269sp**	**39**	**“**	**“**	**“**	**“**	**“**	**“**	**“**	**St. Petersburg**	**Cervicitis**
E/4s	40	6	4	3	3	7	4	3	San Francisco	Cervicitis/urethritis
**Ja/26s**	**41**	**6**	**4**	**3**	**5**	**6**	**4**	**8**	**San Francisco**	**Cervicitis/urethritis**
C/31n	42	7	3	3	1	3	5	7	Nepal	Trachoma, TI
C/35n	43	7	3	3	6	3	5	7	Nepal	Trachoma, TI
C/1n	44	7	3	3	6	3	6	7	Nepal	Trachoma, TI
C/29n	44	“	“	“	“	“	“	“	Nepal	Trachoma, TI
C/30n	44	“	“	“	“	“	“	“	Nepal	Trachoma, TI
C/34n	44	“	“	“	“	“	“	“	Nepal	Trachoma, TI
C/36n	44	“	“	“	“	“	“	“	Nepal	Trachoma, TI
C/37n	44	“	“	“	“	“	“	“	Nepal	Trachoma, TI
F/1-93	45	3	3	3	2	7	4	3	Seattle	Cervicitis
F/6-94	45	“	“	“	“	“	“	“	Seattle	Cervicitis
F/11-96	45	“	“	“	“	“	“	“	Seattle	Cervicitis
**D1/90i**	**45**	**“**	**“**	**“**	**“**	**“**	**“**	**“**	**Indianapolis**	**Cervicitis**
**D1/91i**	**45**	**“**	**“**	**“**	**“**	**“**	**“**	**“**	**Indianapolis**	**Urethra**
**D/13-96**	**45**	**“**	**“**	**“**	**“**	**“**	**“**	**“**	**Seattle**	**Cervix**
**D/199nl**	**45**	**“**	**“**	**“**	**“**	**“**	**“**	**“**	**The Netherlands**	**Cervicitis**
**E/306sp**	**45**	**“**	**“**	**“**	**“**	**“**	**“**	**“**	**St. Petersburg**	**Cervicitis**
E/92i	46	6	3	3	5	7	4	8	Indianapolis	Urethra
E/93i	46	“	“	“	“	“	“	“	Indianapolis	Cervix
E/104i	46	“	“	“	“	“	“	“	Indianapolis	Urethra
E/105i	46	“	“	“	“	“	“	“	Indianapolis	Cervix
E/173i	46	“	“	“	“	“	“	“	Indianapolis	Cervix
E/174i	46	“	“	“	“	“	“	“	Indianapolis	Urethra
E/185i	46	“	“	“	“	“	“	“	Indianapolis	Urethra
E/188i	46	“	“	“	“	“	“	“	Indianapolis	Cervix
**D/210nl**	**46**	**“**	**“**	**“**	**“**	**“**	**“**	**“**	**The Netherlands**	**Cervicitis**
E/100i	47	6	4	3	5	6	4	3	Indianapolis	Cervix
E/101i	47	“	“	“	“	“	“	“	Indianapolis	Urethra
D/EC	48	3	3	3	6	6	4	1	Montana	
D/LC	48	“	“	“	“	“	“	“	Montana	
**K/SotonK1**	**49**	**3**	**3**	**3**	**6**	**6**	**4**	**13**	**United Kingdom**	**Cervicitis**
**B/Jali20**	**50**	**3**	**3**	**3**	**6**	**6**	**5**	**7**	**Gambia**	**Trachoma**
G/11074	51	3	3	3	6	6	9	3	Seattle	Proctitis
G/9301	51	“	“	“	“	“	“	“	Seattle	Urethritis
G/9768	51	“	“	“	“	“	“	“	Seattle	Proctitis
G/11222	52	3	3	5	6	6	1	3	Seattle	Cervicitis
G/272sp	52	“	“	“	“	“	“	“	St. Petersburg	Cervicitis
G/223nl	52	“	“	“	“	“	“	“	The Netherlands	Cervix
G/228nl	52	“	“	“	“	“	“	“	The Netherlands	Cervicitis
**A/HAR13**	**53**	**3**	**5**	**3**	**6**	**3**	**5**	**7**	**Tunisia**	**Trachoma**
F/1	54	6	3	3	2	6	4	3	Seattle	Cervicitis
F/316sp	54	“	“	“	“	“	“	“	St. Petersburg	Cervicitis
F4/175i	55	6	3	3	2	7	4	12	Indianapolis	Cervix
F4/176i	55	“	“	“	“	“	“	“	Indianapolis	Urethra
J/151s	56	6	3	3	5	7	4	3	San Francisco	Cervicitis

L2/208nl	57	1	2	3	8	1	4	11	The Netherlands	PID
L2/246nl	58	3	3	3	8	1	4	11	The Netherlands	PID
G/222nl	59	3	6	5	6	6	1	3	The Netherlands	Cervix
G/224nl	59	“	“	“	“	“	“	“	The Netherlands	Cervix
**D/195nl**	**60**	**6**	**3**	**3**	**9**	**7**	**10**	**8**	**The Netherlands**	**Cervix**
E/212nl	61	6	4	3	2	9	4	3	The Netherlands	Cervix
**D/198nl**	**62**	**6**	**4**	**3**	**5**	**8**	**4**	**8**	**The Netherlands**	**Cervicitis**
**G/226nl**	**63**	**8**	**3**	**3**	**6**	**6**	**8**	**3**	**The Netherlands**	**Cervix**
E/211nl	64	9	4	3	2	7	4	3	The Netherlands	Cervix
**K/291sp**	**65**	**3**	**3**	**3**	**2**	**6**	**8**	**3**	**St. Petersburg**	**Cervicitis**
**B/277sp**	**66**	**3**	**3**	**3**	**2**	**6**	**12**	**18**	**St. Petersburg**	**Cervicitis**
G/270sp	67	3	3	3	6	6	4	15	St. Petersburg	Cervicitis
**B/300sp**	**68**	**3**	**3**	**3**	**6**	**6**	**8**	**6**	**St. Petersburg**	**Cervicitis**
**G/302sp**	**68**	**“**	**“**	**“**	**“**	**“**	**“**	**“**	**St. Petersburg**	**Cervicitis**
**J/283sp**	**69**	**3**	**3**	**3**	**6**	**6**	**8**	**14**	**St. Petersburg**	**Cervicitis**
Ia/262sp	70	3	3	3	6	6	8	16	St. Petersburg	Cervicitis
**E/314sp**	**71**	**3**	**3**	**3**	**6**	**6**	**11**	**3**	**St. Petersburg**	**Cervicitis**
**D/263sp**	**72**	**3**	**3**	**3**	**9**	**7**	**4**	**17**	**St. Petersburg**	**Cervicitis**
**J/259b**	**73**	**3**	**3**	**3**	**10**	**6**	**1**	**6**	**Boston**	**Cervicitis**
**G/308sp**	**74**	**3**	**3**	**3**	**11**	**7**	**4**	**3**	**St. Petersburg**	**Cervicitis**
E/264sp	75	3	4	3	2	7	4	3	St. Petersburg	Cervicitis
E/261sp	76	3	4	3	2	10	4	3	St. Petersburg	Cervicitis
E/265sp	76	“	“	“	“	“	“	“	St. Petersburg	Cervicitis
G/304sp	77	3	4	5	6	6	1	3	St. Petersburg	Cervicitis
**D/297sp**	**78**	**3**	**7**	**3**	**6**	**6**	**4**	**3**	**St. Petersburg**	**Cervicitis**
**D/288sp**	**79**	**6**	**3**	**3**	**9**	**7**	**4**	**8**	**St. Petersburg**	**Cervicitis**
**D/310sp**	**79**	**“**	**“**	**“**	**“**	**“**	**“**	**“**	**St. Petersburg**	**Cervicitis**
E/274sp	80	6	4	3	2	10	4	3	St. Petersburg	Cervicitis
E/287sp	80	“	“	“	“	“	“	“	St. Petersburg	Cervicitis
E/309sp	80	“	“	“	“	“	“	“	St. Petersburg	Cervicitis
E/281sp	81	6	4	8	2	7	4	3	St. Petersburg	Cervicitis
E/282sp	82	10	4	3	2	7	4	3	St. Petersburg	Cervicitis
E/293sp	83	11	4	3	2	7	4	3	St. Petersburg	Cervicitis
**K/254b**	**84**	**12**	**3**	**3**	**2**	**7**	**4**	**3**	**Boston**	**Cervicitis**

Note: **Boldface** denotes putative recombinant

† The strain ID is comprised of the letter that denotes the samples *omp*A genotype (e.g., E) followed by a backslash and then the number of the same that was assigned based on when it was added to the database (e.g., the first samples added have numbers 1, 2, 3, etc while samples added later have higher numbers. The last 1 or 2 letters denote the geographic region from which the sample derived and correlates with the region of isolation (e.g., sp, St. Petersberg; nl, The Netherlands; b, Boston).

Supplementary Table 4. Distribution of STs by geographic region.

ST	Africa n=21	Europe n=109	Russia n=58	Asia n=13	Americas n=122
1	3	13			9
2					1
3					1
4	1				
5		1			
6				1	
7					1
8					1
9					2
10	1				
11				1	
12	12				
13				2	
14					1
15		3	3		6
16					1
17					1
18					1
19		23	13		2
20		1			
21					2
22	1				
23		15	3		14
24					2
25					1
26		1			
27		1			
28		1			
29		1			
30					1
31		1			
32		1			
33		2			
34		20	3		15
35					3
36		1			
37				1	
38					1
39	1	10	10		24
40					1
41					1
42				1	

43				1	
44				6	
45		1	1		6
46		1			8
47					2
48					2
49		1			
50	1				
51					3
52		2	1		1
53	1				
54			1		1
55					2
56					1
57		1			
58		1			
59		2			
60		1			
61		1			
62		1			
63		1			
64		1			
65			1		
66			1		
67			1		
68			2		
69			1		
70			1		
71			1		
72			1		
73					1
74			1		
75			1		
76			2		
77			1		
78			1		
79			2		
80			3		
81			1		
82			1		
83			1		
84					1

Supplementary Table 5: eBURST report for 323 isolates*

Group 1: No. Isolates = 323 | No. STs = 84 | Predicted Founder = 19

ST	FREQ	SLV	DLV	TLV	SAT	Average Distance
19	41	15	24	17	27	2.75
23	32	14	18	15	36	3.2
39	46	10	10	6	57	4.21
34	38	9	13	10	51	3.65
21	2	8	20	23	32	3.13
20	1	7	22	18	36	3.2
75	1	7	7	15	54	3.81
48	2	6	22	19	36	3.22
24	2	6	20	15	42	3.57
68	2	6	20	15	42	3.65
49	1	5	23	19	36	3.24
67	1	5	23	19	36	3.24
32	1	5	8	8	62	4.43
83	1	5	8	8	62	4.43
82	1	5	8	8	62	4.43
64	1	5	8	8	62	4.43
11	1	5	5	23	50	4
51	3	4	27	16	36	3.33
17	1	4	27	16	36	3.33
71	1	4	27	16	36	3.33
70	1	4	21	16	42	3.68
69	1	4	21	16	42	3.68
45	8	4	18	26	35	3.25
15	13	4	16	23	40	3.72
27	1	4	16	21	42	3.59
29	1	4	14	17	48	4.03
56	1	4	11	17	51	3.8
35	3	4	9	16	54	4.02
46	9	4	6	12	61	4.18
8	1	3	19	28	33	3.19
30	1	3	19	19	42	3.57
16	1	3	18	22	40	3.65
31	1	3	14	18	48	4.06
5	1	3	14	18	48	4.06
52	4	3	11	21	48	4.09
36	1	3	11	16	53	3.85
80	3	3	10	9	61	4.46
79	2	3	6	12	62	4.21
43	1	3	4	3	73	4.59
7	1	2	21	27	33	3.15

50	1	2	21	23	37	3.66
9	2	2	14	25	42	3.63
3	1	2	13	29	39	3.48
78	1	2	13	29	39	3.49
84	1	2	13	15	53	3.86
40	1	2	11	13	57	4.42
61	1	2	11	9	61	4.48
55	2	2	10	13	58	4.13
12	12	2	8	23	50	4.03
76	2	2	7	13	61	4.07
13	2	2	5	26	50	4.07
37	1	2	5	13	63	4.27
44	6	2	2	4	75	4.66
59	2	2	1	13	67	4.83
77	1	2	1	13	67	4.66
41	1	2	1	10	70	4.56
57	1	2	1	0	80	5.42
22	1	1	19	24	39	3.72
65	1	1	17	23	42	3.51
10	1	1	14	27	41	3.54
54	2	1	13	31	38	3.46
63	1	1	13	20	49	3.81
25	1	1	12	34	36	3.4
14	1	1	11	28	43	3.57
74	1	1	10	33	39	3.46
81	1	1	10	9	63	5.07
38	1	1	9	12	61	4.5
28	1	1	8	23	51	4.07
47	2	1	7	14	61	4.19
53	1	1	4	5	73	4.73
73	1	1	3	19	60	4.25
62	1	1	2	8	72	5.01
60	1	1	2	6	74	4.79
42	1	1	2	5	75	5.12
1	25	1	2	0	80	5.42
33	2	1	2	0	80	5.43
18	1	0	20	22	41	3.78
26	1	0	6	21	56	4.21
72	1	0	4	21	58	3.92
58	1	0	4	13	66	4.09
2	1	0	3	25	55	3.81
6	1	0	2	7	74	4.6
4	1	0	2	3	78	4.97
66	1	0	1	23	59	4.13

* ST, sequence type; FREQ, frequency; SLV, single locus variant; DLV, double locus variant; TLV, triple locus variant; SAT, satellite

Supplementary Table 6: Evidence of recombination for samples from Amsterdam, The Netherlands.

Sample	*omp*A genotype	ST sequence homology*	ST (majority *omp*A)	Location of SNPs	
L2/237nl	L2	L2	1 (L)		
K/250nl	L2	G or K	15 (K)	G: *pdhA,* 339; *pykF*, 40; *lysS*, 34; *leuS*: 282	K: *pdhA,* 339; *pykF*, 40; *lysS*, 34; *leuS*: 282
**B/193nl**	**B**	**G or K**	**19 (G)**	**G: *pdhA*, 339; *pykF*, 40**	**K: *pdhA*, 339; *pykF*, 40**
**B/194nl**	**B**	**G or K**	**19 (G)**	**G: *pdhA*, 339; *pykF*, 40**	**K: *pdhA*, 339; *pykF*, 40**
**D/202nl**	**D**	**G or K**	**19 (G)**	**G: *pdhA*, 339; *pykF*, 40**	**K: *pdhA*, 339; *pykF*, 40**
**D/203nl**	**D**	**G or K**	**19 (G)**	**G: *pdhA*, 339; *pykF*, 40**	**K: *pdhA*, 339; *pykF*, 40**
**D/204nl**	**D**	**G or K**	**19 (G)**	**G: *pdhA*, 339; *pykF*, 40**	**K: *pdhA*, 339; *pykF*, 40**
**D/205nl**	**D**	**G or K**	**19 (G)**	**G: *pdhA*, 339; *pykF*, 40**	**K: *pdhA*, 339; *pykF*, 40**
**D/206nl**	**D**	**G or K**	**19 (G)**	**G: *pdhA*, 339; *pykF*, 40**	**K: *pdhA*, 339; *pykF*, 40**
**D/207nl**	**D**	**G or K**	**19 (G)**	**G: *pdhA*, 339; *pykF*, 40**	**K: *pdhA*, 339; *pykF*, 40**
G/225nl	G	G or K	19 (G)	G: *pdhA,* 339; *pykF*, 40	K: *pdhA,* 339; *pykF*, 40
G/227nl	G	G or K	19 (G)	G: *pdhA,* 339; *pykF*, 40	K: *pdhA,* 339; *pykF*, 40
G/229nl	G	G or K	19 (G)	G: *pdhA,* 339; *pykF*, 40	K: *pdhA,* 339; *pykF*, 40
G/230nl	G	G or K	19 (G)	G: *pdhA,* 339; *pykF*, 40	K: *pdhA,* 339; *pykF*, 40
**I/240nl**	**I**	**G or K**	**19 (G)**	**G: *pdhA*, 339; *pykF*, 40**	**K: *pdhA*, 339; *pykF*, 40**
**I/241nl**	**I**	**G or K**	**19 (G)**	**G: *pdhA*, 339; *pykF*, 40**	**K: *pdhA*, 339; *pykF*, 40**
**I/245nl**	**I**	**G or K**	**19 (G)**	**G: *pdhA*, 339; *pykF*, 40**	**K: *pdhA*, 339; *pykF*, 40**
**K/248nl**	**K**	**G or K**	**19 (G)**	**G: *pdhA*, 339; *pykF*, 40**	**K: *pdhA*, 339; *pykF*, 40**
**K/249nl**	**K**	**G or K**	**19 (G)**	**G: *pdhA*, 339; *pykF*, 40**	**K: *pdhA*, 339; *pykF*, 40**
**H/231nl**	**H**	**Ia**	**23 (Ia)**		
**H/232nl**	**H**	**Ia**	**23 (Ia)**		

**H/233nl**	**H**	**Ia**	**23 (Ia)**		
**H/234nl**	**H**	**Ia**	**23 (Ia)**		
**I/235nl**	**I**	**Ia**	**23 (Ia)**		
**I/236nl**	**I**	**Ia**	**23 (Ia)**		
**I/238nl**	**I**	**Ia**	**23 (Ia)**		
**I/239nl**	**I**	**Ia**	**23 (Ia)**		
**I/242nl**	**I**	**Ia**	**23 (Ia)**		
**I/243nl**	**I**	**Ia**	**23 (Ia)**		
**I/244nl**	**I**	**Ia**	**23 (Ia)**		
**J/247nl**	**J**	**Ia**	**23 (Ia)**		
**K/251nl**	**K**	**Ia**	**23 (Ia)**		
**D/196nl**	**D**	**F**	**34 (F)**		
**D/197nl**	**D**	**F**	**34 (F)**		
**D/200nl**	**D**	**F**	**34 (F)**		
**D/201nl**	**D**	**F**	**34 (F)**		
**D/209nl**	**D**	**F**	**34 (F)**		
**E/214nl**	**E**	**F**	**34 (F)**		
**J/252nl**	**J**	**F**	**34 (F)**		
F/217nl	F	F	34 (F)		
F/218nl	F	F	34 (F)		
F/219nl	F	F	34 (F)		
F/220nl	F	F	34 (F)		
F/221nl	F	F	34 (F)		
E/213nl	E	E	39 (E)		
E/215nl	E	E	39 (E)		
E/216nl	E	E	39 (E)		
**D/199nl**	**D**	**F**	**45 (F)**	**F: *glyA*, 176; *glyA*, 264; *glyA,* 420**	
**D/210nl**	**D**	**E**	**46 (E)**		
G/223nl	G	G	52 (G)		
G/228nl	G	G	52 (G)		
L2/208nl	L2	L2	57 (L2)	L1, L2, L2a or L3: *mdhC*; 76	
L2/246nl	L2	L2	58 (L2)	L1, L2, L2a or L3: *glyA;* 215; *glyA*: 420; *mdhC*: 76; *mdhC*: 211	
G/222nl	G	G	59 (G)	G: *mdhC:* 194; *lysS:* 34	
G/224nl	G	G	59 (G)	G: *mdhC:* 194; *lysS:* 34	
**D/195nl**	**D**	**Da or F**	**60 (Da)**	**Da: *yhbG*: 267, 369, 451; *pykF*: 317; *lysS*: 280; *leuS*: 58; *leuS*: 58, 96**	**F: *yhbG*: 267, 369, 451; *pykF*: 317; *lysS*: 280; *leuS*: 58; *leuS*: 58, 96**

E/212nl	E	E	61 (E)	E: *pykF*: 484	
**D/198nl**	**D**	**Da or E**	**62 (Da)**	**Da: *yhbG*: 267, 451; *mdhC*: 183; *pykF*: 518, 521; *leuS*: 58; *leuS*: 96**	**E: *yhbG*: 267, 451; *mdhC*: 183; *pykF*: 518, 521; *leuS*: 58; *leuS*: 96**
**G/226nl**	**G**	**G or Ia**	**63 (G)**	**Ia: *glyA*: 191**	
E/211nl	E	E	64 (E)	E: *glyA*: 313	

Note: **Boldface** denotes putative recombinant; Occasionally both the *omp*A genotype and MLST ST match but where there are one or a few SNPs in the 7 genes.
**The seven ST genes of the sample have the highest homology to the seven genes of the strain that has the *omp*A genotype denoted in the column (e.g., for sample H/231nl that has a H *omp*A genotype, the seven ST genes were identical to the seven genes of Ia strains in the database where the *omp*A genotype was also Ia for those strains).

Supplemental Table 7: Evidence of recombination for samples from St. Petersburg, Russia.

**Sample**	***omp*A genotype**	**ST sequence homology**	**ST (majority *omp*A)**	**Location of SNPs**	
E/261sp	E	E	76 (E)		
Ia/262sp	Ia	Ia	70 (Ia)		
**D/263sp**	**D**	**Da or F**	**72 (Da)**	**Da: *glyA:* 176 A→G, 264 C→T, 420 G→A<br>*ybhG*: 451 A→G<br>*leusS*: 58 G→A, 96 G→C, 422 G→A**	**F: *glyA:* 176 A→G, 264 C→T, 420 G→A<br>*ybhG:* 267 G→A<br>*leusS*: 58 G→A, 96 G→C, 422 G→A**
E/264sp	E	E	75 (E)		
E/265sp	E	E	76 (E)		
G/266sp	G	G or K	19 (G)	G: *pdhA*: 339 C→T	K: *pykF*: 40 G→A
**K/267sp**	**K**	**G**	**19 (K)**		
**G/268sp**	**G**	**Ia**	**23 (Ia)**		
**G/269sp**	**G**	**E**	**39 (E)**		
G/270sp	G	G or K	67 (G)	G: *pdhA*: 339 C→T<br>*leusS*: 397 A→G	K: *pykF*: 40 G→A<br>*leusS*: 397 A→G
G/271sp	G	G or K	19 (G)	G: *pdhA*: 339 C→T	K: *pykF*: 40 G→A
G/272sp	G	G	52 (G)		
G/273sp	G	G	19 (G)		
E/274sp	E	E	80 (E)		
E/275sp	E	E	39 (E)		
E/276sp	E	E	39 (E)		
**B/277sp**	**B**	**F**	**66 (F)**		
E/278sp	E	E	39 (E)		
**E/279sp**	**E**	**G or K**	**19 (G)**	**G: *pdhA*: 339 C→T**	**K: *pykF*: 40 G→A**
E/280sp	E	E	39 (E)		
E/281sp	E	E	81 (E)		
E/282sp	E	E	82 (E)		
**J/283sp**	**J**	**Ia**	**69 (Ia)**		
G/284sp	G	G	19 (G)		
E/285sp	E	E	39 (E)		
G/286sp	G	G or K	19 (G)	G: *pdhA*: 1 C→G, 339 C→T	K: *pdhA*: 1 C→G<br>*pykF*: 40 G→A
E/287sp	E	E	80 (E)		
**D/288sp**	**D**	**Da or F**	**79 (Da)**	**Da: *ybhG*: 451 A→G<br>*leusS*: 58 G→A, 96 G→C**	**F: *ybhG*: 267 G→A, 369 A→G<br>*leusS*: 58 G→A**
F/289sp	F	F	34 (F)		
E/290sp	E	E	39 (E)		
**K/291sp**	**K**	**F or Ia**	**65 (F/Ia)**	**F: *glyA*: 176 A→G,**	**Ia: *ybhG*: 267 A→G,**

				**264 C→T, 420 G→A**<br>***pykF*: 317 C→T, 384 G→A**<br>***lysS*: 222 G→A, 261 T→C**	**303 A→G, 367 T→C, 369 G→A, 372 T→C, 387 T→C, 451 A→G**
E/292sp	E	E	39 (E)		
E/293sp	E	E	83 (E)		
**E/294sp**	**E**	**G**	**19 (G)**		
G/295sp	G	G	19 (G)		
K/296sp	K	K	15 (K)		
**D/297sp**	**D**	**G or K**	**78 (G/K)**	**G: *mdhC*: 168 G→A, 183 G→A, 339 C→T**<br>***lysS*: 82 A→T**	**K: *mdhC*: 168 G→A, 183 G→A**<br>***pdhA*: 339 C→T**<br>***lysS*: 82 A→T**
**B/298**	**B**	**Ia**	**23 (Ia)**		
E/299sp	E	E	39 (E)		
**B/300sp**	**B**	**Ia**	**68 (Ia)**		
G/301sp	G	G	19 (G)		
**G/302sp**	**G**	**A or Ba**	**68 (A/Ba)**	**A: *mdhC:* 168 G→A, 183 G→A**<br>***lysS:* 222 G→A**<br>***leuS:* 96 G→C, 282 T→C**	**Ba: *mdhC:* 168 G→A,**<br>**183 G→A**<br>***lysS:* 202 G→A, 222 G→A**<br>***leuS:* 58 A→G**
G/303sp	G	G or I	19 (G)	G: *pdhA*: 339 C→T	I: *pykF*: 40 G→A
G/304sp	G	G	77 (G)		
K/305sp	K	G or K	15 (K)	G: *pdhA*: 339 C→T<br>*lysS*: 34 A→G<br>*leusS*: 282 T→C	K: *pykF*: 40 G→A<br>*lysS*: 34 A→G<br>*leusS*: 282 T→C
**E/306sp**	**E**	**F**	**45 (F)**		
E/307sp	E	E	39 (E)		
**G/308sp**	**G**	**D**	**74 (D)**		
E/309sp	E	E	80 (E)		
**D/310sp**	**D**	**Da or F**	**79 (Da)**	**Da: *ybhG*: 451 A→G**<br>***leusS*: 58 G→A, 96 G→C**	**F: *ybhG*: 267 G→A, 369A→G**<br>***leusS*: 58 G→A**
**J/311sp**	**J**	**Ia**	**23 (Ia)**		
F/312sp	F	F	34 (F)		
F/313sp	F	F	34 (F)		
**E/314sp**	**E**	**G or K**	**71 (G/K)**	**G: *pdhA*: 339 C→T**<br>***lysS*: 221 G→A, 470 G→A**	**K: *pykF*: 40 G→A**<br>***lysS*: 221 G→A, 470 G→A**
G/315sp	G	G	19 (G)		
F/316sp	F	F	54 (F)		

G/317sp	G	G or K	19 (G)	G: *pdhA*: 339 C→T<br>*lysS*: 34 A→G	K: *pykF*: 40 G→A<br>*lysS*: 34 A→G
**J/318sp**	**J**	**G or K**	**15 (G/K)**	**G: *pdhA*: 339 C→T<br>*lysS*: 34 A→G**	**K: *pykF*: 40 G→A<br>*lysS*: 34 A→G**

Note: **Boldface** denotes putative recombinant
*The seven ST genes of the sample have the highest homology to the seven genes of the strain that has the *omp*A genotype denoted in the column (e.g., for sample G/268sp that has a G *omp*A genotype, the seven ST genes were identical to the seven genes of Ia strains in the database where the *omp*A genotype was also Ia for those strains).

Supplementary Table 8: Pairwise Population Differentiation (Fst) for North American, Dutch, and Russian Women

Comparison	Fst
American vs. Netherlands Women	0.05332*
Russian vs. American Women	0.00966 ns
Russian vs. Netherlands Women	0.01996 ns
Russian vs. Netherlands Women non-LGV	0.01101 ns
American vs Netherlands Women non-LGV L2	0.04671*

*$p < 0.05$ by permutation test ($n = 110$)

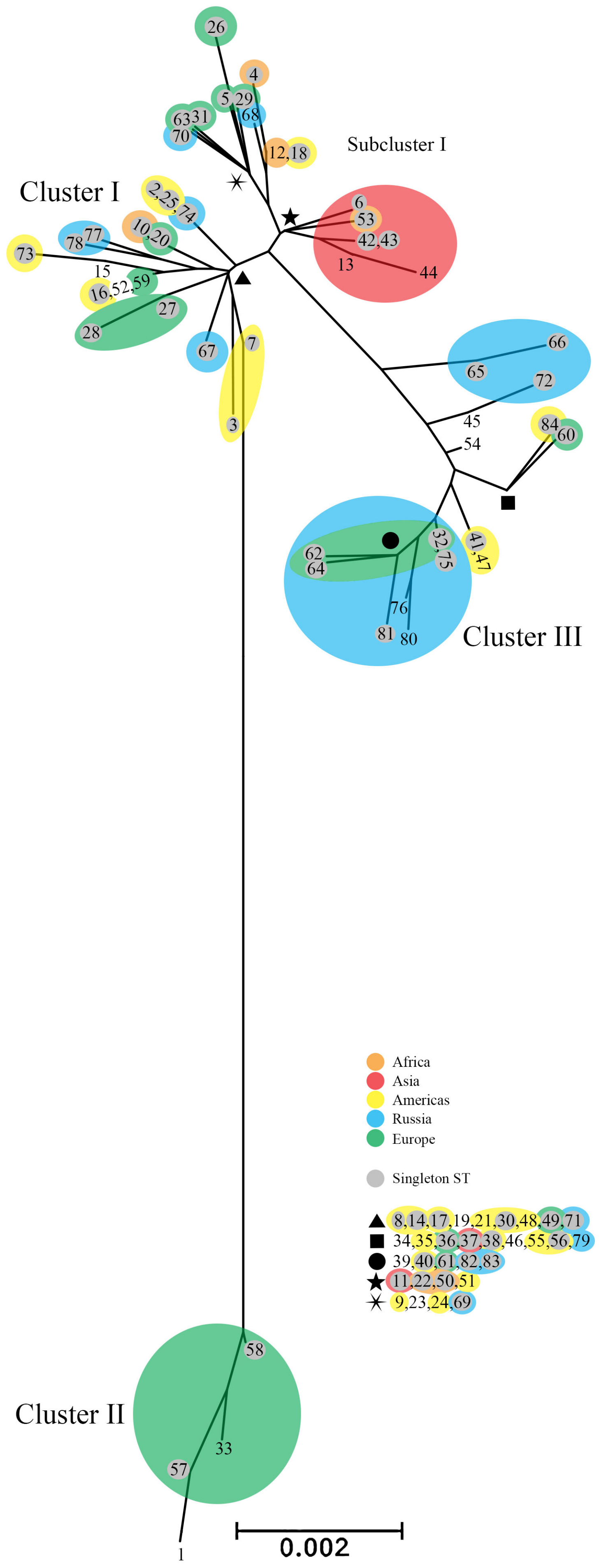
Cluster I
Subcluster I
Cluster II
Cluster III
0.002
Africa
Asia
Americas
Russia
Europe
Singleton ST
▲ 8,14,17,19,21,30,48,49,71
■ 34,35,36,37,38,46,55,56,79
● 39,40,61,82,83
★ 11,22,50,51
✶ 9,23,24,69

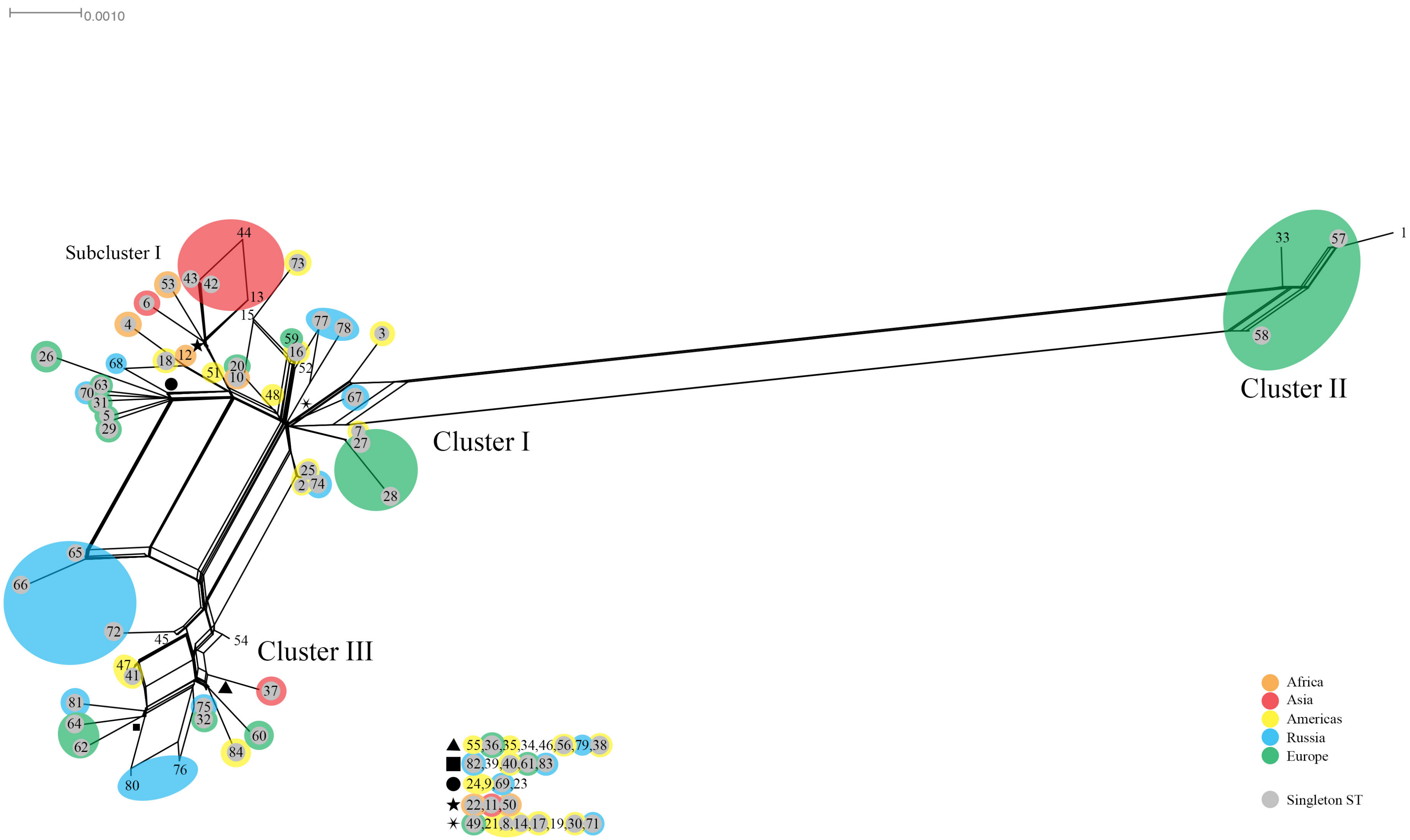

0.0010
Subcluster I
Cluster I
Cluster II
Cluster III
▲ 55,36,35,34,46,56,79,38
■ 82,39,40,61,83
● 24,9,69,23
★ 22,11,50
✶ 49,21,8,14,17,19,30,71
Africa
Asia
Americas
Russia
Europe
Singleton ST
